# Phytochrome-Mediated Light Perception Affects Fruit Development and Ripening Through Epigenetic Mechanisms

**DOI:** 10.3389/fpls.2022.870974

**Published:** 2022-04-28

**Authors:** Ricardo Bianchetti, Nicolas Bellora, Luis A. de Haro, Rafael Zuccarelli, Daniele Rosado, Luciano Freschi, Magdalena Rossi, Luisa Bermudez

**Affiliations:** ^1^Departamento de Botânica, Instituto de Biociências, Universidade de São Paulo, São Paulo, Brazil; ^2^Institute of Nuclear Technologies for Health (Intecnus), National Scientific and Technical Research Council (CONICET), Bariloche, Argentina; ^3^Department of Plant and Environmental Sciences, Weizmann Institute of Science, Rehovot, Israel; ^4^Instituto de Agrobiotecnología y Biología Molecular (IABIMO), CICVyA, INTA-CONICET, Castelar, Argentina; ^5^Cátedra de Genética, Facultad de Agronomía, Universidad de Buenos Aires, Buenos Aires, Argentina

**Keywords:** carotenoid, chlorophyll, DNA methylation, epigenetics, fleshy fruit, RdDM, tomato

## Abstract

Phytochrome (PHY)-mediated light and temperature perception has been increasingly implicated as important regulator of fruit development, ripening, and nutritional quality. Fruit ripening is also critically regulated by chromatin remodeling *via* DNA demethylation, though the molecular basis connecting epigenetic modifications in fruits and environmental cues remains largely unknown. Here, to unravel whether the PHY-dependent regulation of fruit development involves epigenetic mechanisms, an integrative analysis of the methylome, transcriptome and sRNAome of tomato fruits from *phyA* single and *phyB1B2* double mutants was performed in immature green (IG) and breaker (BK) stages. The transcriptome analysis showed that PHY-mediated light perception regulates more genes in BK than in the early stages of fruit development (IG) and that PHYB1B2 has a more substantial impact than PHYA in the fruit transcriptome, in both analyzed stages. The global profile of methylated cytosines revealed that both PHYA and PHYB1B2 affect the global methylome, but PHYB1B2 has a greater impact on ripening-associated methylation reprogramming across gene-rich genomic regions in tomato fruits. Remarkably, promoters of master ripening-associated transcription factors (TF) (*RIN*, *NOR*, *CNR*, and *AP2a*) and key carotenoid biosynthetic genes (*PSY1*, *PDS*, *ZISO*, and *ZDS*) remained highly methylated in *phyB1B2* from the IG to BK stage. The positional distribution and enrichment of TF binding sites were analyzed over the promoter region of the *phyB1B2* DEGs, exposing an overrepresentation of binding sites for RIN as well as the PHY-downstream effectors PIFs and HY5/HYH. Moreover, *phyA* and *phyB1B2* mutants showed a positive correlation between the methylation level of sRNA cluster-targeted genome regions in gene bodies and mRNA levels. The experimental evidence indicates that PHYB1B2 signal transduction is mediated by a gene expression network involving chromatin organization factors (DNA methylases/demethylases, histone-modifying enzymes, and remodeling factors) and transcriptional regulators leading to altered mRNA profile of ripening-associated genes. This new level of understanding provides insights into the orchestration of epigenetic mechanisms in response to environmental cues affecting agronomical traits.

## Introduction

As sessile organisms, plants must constantly monitor their environment and continuously tune their gene expression to enable adaptation and survival (Kaiserli et al., 2018). Light is one of the primary environmental cues that controls plant growth and development from seed germination to senescence (Galvão and Fankhauser, 2015). Plants employ different photoreceptors to detect and respond to changes in the incident spectral composition (from UV-B to far-red wavelengths), light direction and photoperiod. These photoreceptor families include (i) phytochromes (PHYs), which perceive red/far-red (R/FR) light; (ii) cryptochromes (CRYs), phototropins, and “Zeitlupes,” which sense blue/UV-A light; and (iii) the UV-B receptor UVR8 (Paik and Huq, 2019).

After photoreceptor activation, complex signal transduction pathways control the expression of light-regulated genes *via* transcriptional, posttranscriptional, and posttranslational mechanisms (Galvão and Fankhauser, 2015). Several hub proteins in the light signal transduction pathway triggered by PHYs, CRYs and UVR8 have been identified, including transcription factors (TFs) such as PHY-INTERACTING FACTORS (PIFs) and ELONGATED HYPOCOTYL5 (HY5), HY5-HOMOLOGUE (HYH), as well as the ubiquitin E3 ligase complexes comprising CONSTITUTIVE PHOTOMORPHOGENIC1 (COP1) (Galvão and Fankhauser, 2015). Yet, PHYA and PHYB can directly bind to target promoters (Chen et al., 2014; Jung et al., 2016) and, recently, the effect of light on alternative splicing (AS) has also been reported (Shikata et al., 2014; Cheng and Tu, 2018). Furthermore, light controls protein localization through PHY-mediated alternative promoter selection, allowing plants to metabolically respond to changing light conditions (Ushijima et al., 2017). Finally, it is widely known that activated PHYs induce post-translational changes in PIF proteins, including sequestration, phosphorylation, polyubiquitylation, and subsequent degradation through the 26S proteasome-mediated pathway (Paik and Huq, 2019). In tomato (*Solanum lycopersicum*), five-PHY encoding genes; *PHYA*, *PHYB1*, *PHYB2*, *PHYE*, and *PHYF* have been identified (Alba et al., 2000a), their expression profile is variable along different tissues being *PHYA* and *PHYB2* the most abundant in fruits and, particularly, *PHYB2* is induced along ripening (Supplementary Figure 1). In concordance, evidence suggests that the distinct PHYs play specific functions in different organs. *PHYA* participates in the regulation of carbon metabolic processes, especially in dark-grown seedlings (Carlson et al., 2019), whereas *PHYB1* regulates multiple processes during seedling development (van Tuinen et al., 1995; Weller et al., 2000). Moreover, *PHYB2* is highly expressed in fruits and marked accumulation of *PHYA* has also been verified during ripening (Alba et al., 2000b; Bianchetti et al., 2018). Finally, *PHYE* functions are related to shade avoidance responses (Schrager-Lavelle et al., 2016), while specific roles for *PHYF* remains elusive (Mereb et al., 2020). Tomato *phyA*, *phyB1*, and *phyB2* knockout mutants have been well characterized over the last two decades and several phenotypes associated with light perception deficiency in seedlings and vegetative organs have been described (Kerckhoffs et al., 1996, 1999; Behringer and Lomax, 1999; Weller et al., 2000; Bianchetti et al., 2020). Particularly in fruits, the most abundantly described phenotype is that, in the red ripe stage, fruits with *phyA*, *phyB1* and *phyB2* knockout mutations are impaired in ripening-associated carotenoid accumulation (Kendrick et al., 1997; Behringer and Lomax, 1999; Weller et al., 2000; Bianchetti et al., 2020). Remarkably, the effect of PHYs in carotenogenesis is conserved in both vegetative tissues of *Arabidopsis thaliana* and tomato fruits. By a virtually identical mechanism: PIFs, direct downstream interactors of PHYs, bind to the promoter of the *PHYTOENE SYNTHASE* (*PSY*) gene repressing its expression and limiting carotenogenesis (Toledo-Ortiz et al., 2010; Llorente et al., 2016). Moreover, through different approaches, other studies have shown that PHYA, PHYB1, and PHYB2 positively influence tomato plastid division and significantly impact the accumulation of different nutraceutical compounds in the tomato fruits (Alba et al., 2000a; Schofield and Paliyath, 2005; Bianchetti et al., 2018, 2020; Gramegna et al., 2019; Alves et al., 2020).

Although the effect of light on plant phenotypes and the plant transcriptome has been studied for decades (Mazzella et al., 2005; Ibarra et al., 2013; Carlson et al., 2019), the involvement of epigenetic regulatory mechanisms in light-dependent changes in the transcriptional landscape remains poorly addressed. Post-translational histone modifications, such as acetylation and methylation, have been associated with the induction and repression of light-responsive genes (Tessadori et al., 2009; Perrella and Kaiserli, 2016). Light-dependent enrichment of the acetylation pattern of Histones 3 and 4 (H3, H4) in the enhancer and promoter regions of the pea plastocyanin locus *PetE* has been reported (Chua et al., 2001), which, in turn, activates the transcription of this gene (Chua et al., 2003). Moreover, a reduction in H3 acetylation is associated with a decrease in the expression of the *A. thaliana* light-responsive genes *CHLOROPHYLL a/b-BINDING PROTEIN* (*CAB2*) and the *RIBULOSE BISPHOSPHATE CARBOXYLASE/OXYGENASE* small subunit (*RBCS*) (Bertrand et al., 2005). Histone methylation regulates PHY-mediated seed germination in *A. thaliana*. Upon R light illumination, photoactivated PHYB targets PIF1 for proteasome-mediated degradation, releasing the expression of the *JUMONJI HISTONE DEMETHYLASES* (*JMJ20* and *JMJ22*). As a result, JMJ20 and JMJ22 reduce methylation levels on H4, which leads to the activation of the gibberellic acid biosynthesis pathway to promote seed germination (Cho et al., 2012). Recently, it has been demonstrated that, in the presence of light, PHY-downstream effector HY5 recruits HISTONE DEACETYLASE 9 (HDA9) to autophagy-related genes to repress their expression by deacetylation of H3. In the darkness, HY5 is degraded *via* 26S proteasome and the concomitant disassociation of HDA9 leads to activated autophagy (Yang et al., 2020). Moreover, ChIP-seq studies have revealed that many genes targeted by HY5 are enriched for specific histone marks (Charron et al., 2009).

Together with histone modification, DNA methylation is a common epigenetic mark with a direct impact on gene expression. Nevertheless, only a few reports have specifically addressed the effect of light stimuli on DNA methylation. Light-dependent nuclear organization dynamics during deetiolation is associated with a reduction in methylated DNA (Bourbousse et al., 2020). In *Populus nigra*, 137 genes were shown to be regulated by methylation during the day/night cycle (Ding et al., 2018). Moreover, photoperiod-sensitive male sterility is regulated by RNA-directed DNA methylation (RdDM) in rice (Ding et al., 2012). In tomato, plants overexpressing UV-DAMAGED DNA BINDING PROTEIN 1 (DDB1), a component of the ubiquitin E3 ligase complex, showed reduced size in reproductive organs (flowers, seeds and fruits) associated with the promoter hypomethylation and the upregulation of the cell division negative regulator *WEE1* (Liu et al., 2012). Finally, using a methylation-sensitive amplified polymorphism assay, DNA methylation remodeling was shown to be an active epigenetic response to different light qualities in tomato seedlings (Omidvar and Fellner, 2015).

It is well known that epigenome reprogramming, including DNA methylation status and histone marks, governs fruit ripening, occurring as a developmental switch to restrict the activities of ripening regulators (Zhong et al., 2013; Liu et al., 2015). Ripening-associated epigenetic changes have been shown to be widespread in climacteric, non-climacteric and, even in species bearing dry fruits (Lü et al., 2018). However, only in tomato, genetic evidence links DNA demethylation to a regulatory role in ripening (Zhong et al., 2013; Liu et al., 2015; Lang et al., 2017). Here, to enlighten the regulatory mechanism underneath the role of PHYs on fruit ripening, genome-wide transcriptome, sRNAome and methylome were comprehensively analyzed in fruits from tomato *phyA* and *phyB1B2* null mutants. The results revealed that PHY-mediated gene expression regulation throughout fruit development and ripening involves DNA methylation regulatory machinery.

## Materials and Methods

### Plant Material, Growth Conditions, and Sampling

Seeds of tomato single *phyA* and double *phyB1B2* mutants, in Moneymaker background, were provided by Rameshwar Sharma (University of Hyderabad, India) and their genetic backgrounds were previously characterized (Kerckhoffs et al., 1996, 1999; Lazarova et al., 1998).

Plants were grown in a glasshouse at the Instituto de Biociências, Universidade de São Paulo, 23°33′55″S 46°43′51″W. Tomato seeds were grown in 9 L pots containing a 1:1 mixture of commercial substrate and expanded vermiculite, supplemented with 1 g L^−1^ of NPK 10:10:10, 4 g L^−1^ of dolomite limestone (MgCO_3_ + CaCO_3_) and 2 g L^−1^ thermophosphate in an average mean temperature of 25/18°C (day/night), ~16 h light hours, and 250–350 μmol photons m^−2^ s^−1^ irradiation and relative humidity of 55%. Five plants per genotype were cultivated. All fruits were harvested at the same time of day (between 12 and 13 h) with four biological replicates (each replicate was composed of a complete single fruit from different plants). The columella, placenta, and seeds were immediately removed, and the all the remaining tissues were frozen in liquid nitrogen, ground and kept at −80°C until processing.

Fruits were *a priori* sampled at the same developmental stage instead of necessarily the same age (Supplementary Figure 2A). Fruits were sampled at the immature green (IG, 15 mm diameter, 15 days after anthesis), mature green (MG, when the placenta displays a jelly aspect), breaker (BK, beginning of ripening process when the fruit shows the first yellowish coloration) and red ripe (RR, 7 days after the breaker stage) stages (Supplementary Figure 2A). All three genotypes reached MG and BK stages at approximately 39 and 42 days after anthesis (DAA), respectively, in the above-mentioned growing conditions. To ensure that the fruits collected were at the same physiological stage, the fruit surface color was determined at the equator of each collected fruit using a colorimeter (Konica Minolta, CR-400, 8-mm aperture, D65 illuminant, United States), as described in Cruz et al. (2018). Three measurements were taken at the equator of each fruit and average values were calculated. Hue angle values revealed that fruit color changes show the same progression for the three genotypes with a slight acceleration from BK to RR in the *phyB1B2* mutant, indicating that the fruit collected were at the same physiological stage (Supplementary Figure 2C).

### Transcriptional Profile

Total RNA was extracted from fruits at the immature green and breaker stages with three independent biological replicates of each genotype using a Promega ReliaPrep RNA tissue kit according to the manufacturer’s instructions. The RNA concentration was determined with a spectrophotometer (Nanodrop ND-1000; NanoDrop Technologies, Wilmington, DE, United States), RNA quality was assessed with a BioAnalyzer 2,100 (Agilent Technologies), and RNA libraries were constructed following the recommendations of an Illumina Kit (Directional mRNA-Seq Sample Preparation) and sequenced using the Illumina NovaSeq 6,000 System. Each library was sequenced, generating approximately 20 million 150 bp paired-end reads per sample. The raw sequencing reads were analyzed with FastQC^1^ and, filtered and cleaned using Trimmomatic (Bolger et al., 2014) (Parameters: ILLUMINACLIP: TruSeq3-PE.fa:2:30:10LEADING:3 TRAILING:3 SLIDINGWINDOW:4:20 MINLEN:50). At least 95% (19.1–27.9 M) of the reads met the quality criteria and were mapped to the tomato reference genome sequence SL3.0 with the ITAG3.2 annotation using STAR v2.4.2. allowing one mismatch (Dobin et al., 2013), approximately 84% of the reads were uniquely mapped (Supplementary Table 1) and were used for statistical analysis.

### Reverse Transcription and Quantitative PCR

Total RNA extraction was performed with the ReliaPrep™ RNA Cell and Tissue Miniprep System (Promega), and cDNA synthesis was conducted with SuperScript™ IV Reverse Transcriptase (Invitrogen). The primers used for qPCR are listed in Supplementary Table 2. RT-qPCR was performed in a QStudio6—A1769 PCR Real-Time thermocycler using 2X Power SYBR Green Master Mix in a final volume of 10 μl. Absolute fluorescence data were analyzed using LinRegPCR software to obtain Ct and primer efficiency values. Relative mRNA abundance was calculated and normalized according to the ΔΔCt method using *EXPRESSED* and *CAC* as reference genes (Expósito-Rodríguez et al., 2008).

### MethylC-Seq Analysis

For each genotype, a single gDNA (~5 μg) sample was extracted from a pool of the same three biological replicates used in the transcriptome analyses using the DNeasy Plant maxi kit (Qiagen). The libraries were prepared with the EZ DNA Methylation-Gold Kit (Zymo Research) and the Accel-NGS® Methyl-Seq DNA Library Kit (Swift Biosciences) and further sequenced using the Illumina NovaSeq 6,000 platform. Over 240 M reads were sequenced from each genotype and stage. Raw reads were screened for quality using Trimmomatic (Bolger et al., 2014) (parameters: ILLUMINACLIP:TruSeq3-PE.fa:2:30:10 LEADING:3 TRAILING:3 SLIDINGWINDOW:4:20 MINLEN:50). Mapping to the tomato reference genome sequence SL3.0 and the assessment of global methylation status were performed using Bismark (Krueger and Andrews, 2011) (parameters: bismark -q -bowtie2 -non_directional -N 1 -p. 4), and the methylation status of DNA in the three possible contexts (CG, CHG, and CHH) (H = C, A or T) was distinguished. At least 130 M reads were uniquely mapped (Supplementary Table 3). The Bioconductor package methylKit (Akalin et al., 2012) was used for the detection of methylation levels across the analyzed regions: promoters (2 kb upstream of transcription start site) and sRNA cluster-targeted genome regions (sCTGRs). Only Cs with 10X coverage were considered for these analyses. Methylation differences with a false discovery rate (FDR) < 0.05 in each comparison (WT vs. *phyA*; WT vs. *phyB1B2*) were recorded as differentially methylated promoters (DMPs) or differentially methylated sCTGRs. Differential methylation in the CG, CHG, and/or CHH context was considered if the region contained at least 10 differentially methylated Cs in the corresponding context. Finally, to compare global methylation levels between genotypes, only common Cs with at least 10X coverage in all samples were analyzed.

### sRNAome Profile

sRNA extraction and quality parameters were determined from the same replicates used for transcriptional profile. After RNA integrity confirmation, libraries were prepared using a TruSeq Small RNA Library Prep and sequenced using the Illumina HiSeq 4,000 platform to generate a read length of 50 bp. The raw sequencing reads that were generated were quality trimmed with Trimmomatic (Bolger et al., 2014) to retain reads of 18–24 nt in length (parameters: ILLUMINACLIP:TruSeq3-SE:2:30:10 LEADING:3 TRAILING:3 SLIDINGWINDOW:4:15 MINLEN:18 AVGQUAL:25). A minimum of 38% (WT/breaker/A) and a maximum of 85% (WT/immature green/A) of the reads achieved the quality criteria and were used for further analyses (Supplementary Table 4A). All libraries were aligned to genome version SL3.0 using ShortStack v3.8.1 (Axtell, 2013) with default parameters (allowing the distribution of multimapping reads according to the local genomic context). Then, the *de novo* identification of clusters of sRNAs was performed for all libraries, and individual counts for each library and cluster were obtained using the same software.

### Statistical Analysis for RNAseq and sRNAome

Genes/sRNA clusters with read/count numbers smaller than two per million were removed. Read/count values were normalized according to the library size factors. Statistical analyses were performed with edgeR from Bioconductor® (Robinson et al., 2009; McCarthy et al., 2012) using a genewise negative binomial generalized linear model with the quasi-likelihood test (Chen et al., 2016) and a cutoff of the FDR ≤ 0.05.

### Gene Functional Categorization

The DEGs were functionally categorized with MapMan application software (Thimm et al., 2004), followed by hand-curated annotation using MapMan categories.

### *In silico* Regulatory Motif Predictions and RIN ChIP-Seq Analyses

RIN ChIPseq reads were downloaded from the Sequence Read Archive (SRA) (accession SRX15083; Zhong et al., 2013), mapped to tomato genome version SL3.0 with STAR (Dobin et al., 2013) (version 2.7.3X, parameters: outFilterMismatchNmax 3, alignEndsType EndToEnd, alignIntronMax 5), and peak calling was performed using Macs2 (Zhang et al., 2008) (version 2.2.7.1, default parameters). Regions of 200 bp centred on the top-scoring peaks (score > 100, *n* = 327) were retrieved and the binding motif was inferred *de novo* by using the MEME algorithm (Bailey et al., 2015).

In order to analyze the relative abundance of light regulation associated *cis*-elements, their position frequency matrices (PFM) were retrieve from JASPAR 2020 database (Fornes et al., 2019) for PIFs and HYx (HY5 and HYH) and; from the peak calling of ChIPseq data for RIN (Zhong et al., 2013). The PFMs were scanned with Fimo (Bailey et al., 2015), value of *p* < 1e^−5^ along SL3.0 genome. A 20 Kb region upstream the transcription start site (TSS) was examined for the presence of the TFBSs (Transcriptional Factor Binding Sites). The association was calculated from the accumulative number of genes harbouring a determined *cis*-regulatory element in a specific set of regulated genes, against whole genome random expectation. The signal-to-noise ratio for each position was calculated as the enrichment score (ES) subtracting the regulated genes-set to all annotated promoters. Later, an associated z-score and value of *p* for each class of TF were obtained from the ES distribution of 1,000 random samples set.

### Carotenoid and Chlorophyll Analysis

Chlorophyll, phytoene, phytofluene, lycopene, β-carotene and lutein levels were extracted and determined *via* HPLC with a photodiode array detector as previously described by Lira et al. (2017).

### Statistical Analysis of RT-qPCR and Metabolites

Statistical analyses of the RT-qPCR (Student’s *t*-test, *p* ≤ 0,05) and metabolic data (ANOVA, Tukey’s test. *p* ≤ 0,05) were performed with InfoStat/F software.^2^

### Availability of Data and Materials

The datasets generated and/or analyzed during the current study are available in the Sequence Read Archive (SRA) under NCBI Bioproject PRJNA646733, with accession numbers SUB7763724, SUB7782168 and SUB7791358 for RNAseq, WGBS and small RNAseq, respectively.^3^

## Results

### Impact of Light Perception Impairment on the Fruit Transcriptome

**Figure 1:**
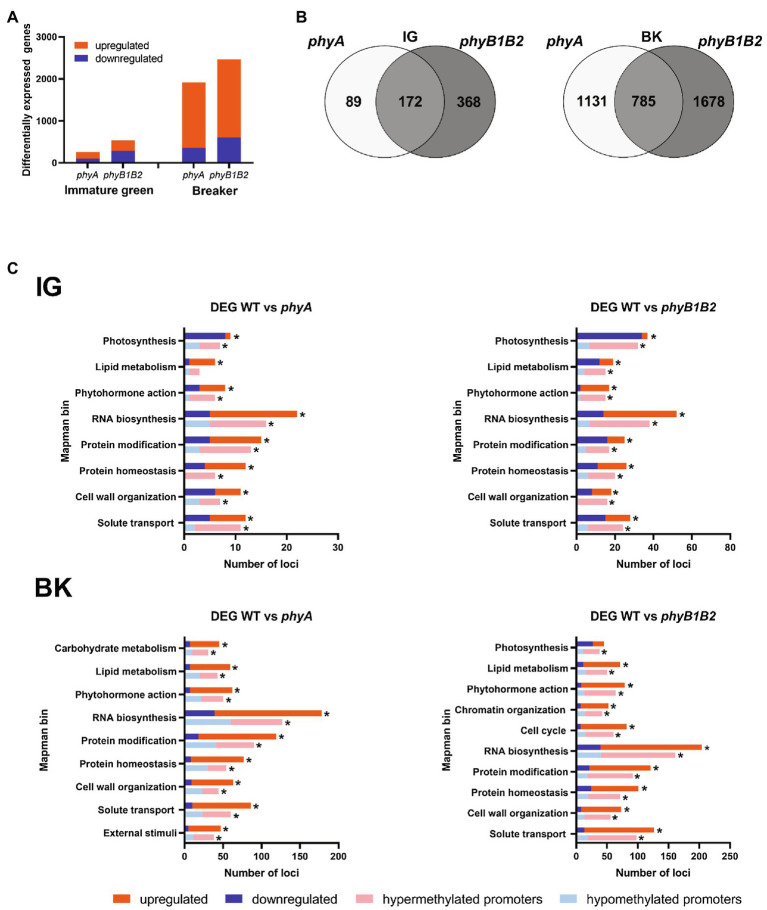
PHYA and PHYB1B2 modify the global transcriptomic profile of tomato fruit. **(A)** Number of differentially expressed genes (DEGs) in *phyA* and *phyB1B2* mutant fruits at immature green (IG) and breaker (BK) stages. **(B)** Venn diagram showing exclusive and common DEGs in *phyA* and *phyB1B2* mutants in both developmental stages. **(C)** Functional categorization of all DEGs and those DEGs with differentially methylated promoters (DMPs) in both analyzed genotypes and stages. Only categories containing at least 2% of the DEGs or DMPs in each comparison are shown (asterisks). Up- and downregulated genes are indicated in red and blue, respectively. Loci with hyper- and hypomethylated promoters are indicated in light red and light blue, respectively. DEGs and DMPs show statistically significant differences (FDR < 0.05) relative to WT.

To investigate the role played by either PHYA or PHYB1 and PHYB2 (hereafter PHYB1B2) in overall gene expression during fruit development, the transcriptome of fruits at the immature green (IG) and breaker (BK) stages from *phyA* and *phyB1B2* null mutants as well as their wild-type (WT) counterpart was determined by RNAseq. Among the approximately 20,000 transcriptionally active loci in each biological replicate (Supplementary Table 1), 1.2% and 2.4% at the IG stage and 9.1% and 11.2% at the BK stage were identified as differentially expressed genes (DEGs) in *phyA* and *phyB1B2* mutants, respectively, compared to the WT (Figure 1A; Supplementary Table 5). For both genotypes, the number of exclusive DEGs was significantly lower in the IG stage than in the BK stage; similarly, the number of genes that were commonly regulated by PHYA and PHYB1B2 was 172 at the IG stage and 785 at the BK stage (Figure 1B). Subsequently, the altered expression of approximately 76% (23/30) of the tested genes was validated by RT-qPCR (Supplementary Table 6). Comparison with previously reported expression data for genes involved in fruit ripening, ethylene biosynthesis and signaling as well as carotenogenesis further validated our RNAseq data, as 90% of the analyzed genes on average showed the expected mRNA profile at IG and BK stages. It is worth mentioning that most of the genes displayed the same transcript fluctuation in the WT, *phyA* and *phyB1B2* genotypes, though this was somewhat attenuated in the mutants (Supplementary Table 7). These results showed that PHY-mediated light perception regulates more genes in BK than in the early stages of fruit development and that PHYB1B2 has a more substantial impact than PHYA in the transcriptome in both analyzed fruit development stages.

A closer look at DEGs function revealed a similar distribution of loci across MapMan categories in response to *phyB1B2* and *phyA* mutations in both developmental stages, although with distinct abundance levels (Figure 1C). At the IG stage, eight categories were mainly represented, including at least 2% of the DEGs identified in *phyA* and *phyB1B2*: photosynthesis, lipid metabolism, phytohormone action, RNA biosynthesis, protein modification, protein homeostasis, cell wall organization, and solute transport (Figure 1C; Supplementary Tables 8 and 9). It is worth highlighting the abundance of the DEGs within the photosynthesis category in the *phyB1B2* mutant, among which 34 out of the 37 DEGs were downregulated (Supplementary Table 9), which is in agreement with the impact of PHY-mediated light perception on chlorophyll metabolism (Kerckhoffs et al., 1999). In the BK stage, at least 2% of the DEGs were related to lipid metabolism, phytohormone action, RNA biosynthesis, protein modification and homeostasis, cell wall organization and solute transport categories in both genotypes (Figure 1C; Supplementary Tables 10 and 11). However, while *phyA* deficiency also affected carbohydrate metabolism and external stimuli (Supplementary Table 10), the *phyB1B2* mutant showed a large number of DEGs related to the cell cycle and chromatin organization (Supplementary Table 11). Interestingly, the chromatin organization category displayed 52 DEGs, 45 of which were upregulated. These genes encode nucleosome constituent histones (H3, H4, H2A, and H2B); DNA methylases/demethylases; histone post-translational modifiers, such as deacetylases, methylases/demethylases, histone ubiquitination factors and histone chaperones; chromatin remodeling factors; and genes involved in RNA-independent and RNA-directed DNA methylation (Supplementary Table 11). These results led us to investigate further the impact of DNA methylation on PHY-mediated gene expression reprogramming.

### PHYs-Dependent Reprogramming of Tomato Fruit Methylome

The global profile of methylated cytosines (mCs) in the epigenome of tomato fruits was assessed by whole-genome bisulfite sequencing in the IG and BK stages for *phyA*, *phyB1B2* and WT genotypes. In agreement with previous reports (Zhong et al., 2013; Zuo et al., 2020), regardless of the genotype and fruit stage, the greatest total number of mCs was located in the CHH context, followed by the CG and CHG contexts, while the methylation level was highest in the CG (80%) context followed by the CHG (67%) and CHH (23%) contexts (Supplementary Table 3; Supplementary Figure 3). For further comparisons, we selected only cytosines with coverage >10X, and except for chromosome 9 in the transposable elements (TEs) enriched region, all samples met this cutoff. In all contexts, the highest cytosine density was associated with gene-rich euchromatic regions located at chromosome arm ends (Supplementary Figure 3). Conversely, in symmetrical contexts (CG and CHG), the highest methylation rates were found across pericentromeric regions enriched in TEs. Yet, the highest methylation rates in CHH context was observed in gene-rich regions associated with a higher density of sRNAs (Supplementary Figure 3), as previously reported (Corem et al., 2018). The comparison of the methylation status between the two fruit stages showed that ripening-associated demethylation (Zhong et al., 2013) occurs mainly in the CG context, especially in gene-rich regions, and that it is impaired in *phyB1B2* mutant BK fruits (Supplementary Figure 3).

**Figure 2:**
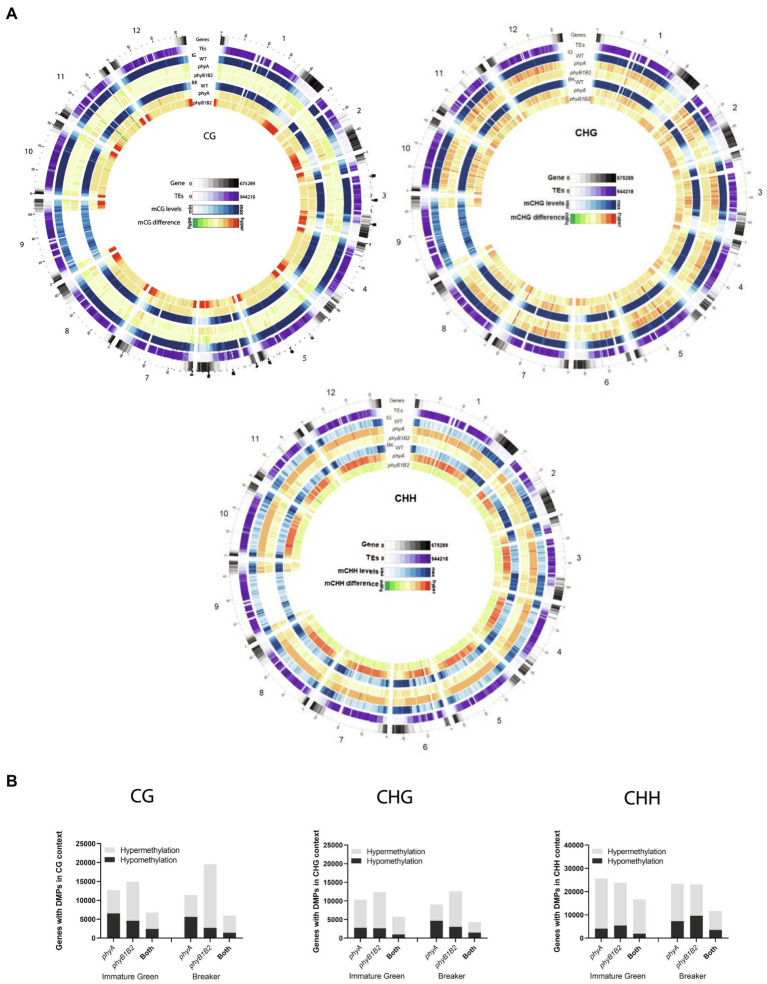
Disturbed PHYA- and PHYB1B2-dependent signaling differentially alters tomato fruit methylome. **(A)** Density plot of genes, transposable elements (TEs) and mC in all contexts (mCG, mCHG, and mCHH) for the wild type (WT) genotype. Global methylation changes for *phyA* and *phyB1B2* in comparison with the WT at the immature green (IG) and breaker (BK) stages are shown (bin size, 1 Mb). Gene and TE densities were estimated according to the number of nucleotides covered per million. The methylation levels in the CG, CHG and CHH contexts are 40%–90%, 25%–80%, and 10%–30%, respectively. The mC difference is relative to the corresponding WT fruit stage within a −5% (hypomethylated) ≤ range ≤ +5% (hypermethylated). Chromosome scale (Mb) is shown. **(B)** Number of genes with differentially methylated promoters (DMPs, 2 kb upstream transcription start site) in *phyA*, *phyB1B2* and common in both mutants, compared to WT. Hyper- and hypomethylation are indicated by grey and darker-colored bars, respectively. DMPs show statistically significant differences (FDR < 0.05) relative to WT.

The subsequent comparison between genotypes revealed global epigenome alteration in *phy* mutants in all methylation contexts analyzed. The most remarkable observation was the presence of considerable hypermethylation in all contexts across gene-rich regions in BK-stage fruits from *phyB1B2* (Figure 2A). In contrast, *phyA* exhibited hypermethylation in CHG and CHH contexts associated with TE-rich regions (Figure 2A), suggesting that different PHYs control DNA methylation across specific genomic regions through distinct regulatory mechanisms. Interestingly, PHY-associated hypomethylation was exclusively detected in the CG context of gene-rich regions in IG-stage fruits from *phyA* and in the CHH context of TE-rich regions for BK-stage fruits from *phyB1B2*. In summary, these data revealed that both PHYA and PHYB1B2 affect the global methylome, but PHYB1B2 has a greater impact on ripening-associated methylation reprogramming across gene-rich genomic regions in tomato fruits.

To investigate the relationship between PHY-dependent modifications in cytosine methylation and gene expression, we first identified genes with differentially methylated promoters compared to WT (DMPs, 2 kb upstream the TSS) in all three methylation contexts. Interestingly, associated with the massive alteration previously observed, the pattern of DMPs varied with the mC context, stage and genotype (Figure 2B; Supplementary Tables 12–16). Regarding the CG context, whereas the *phyA* mutant showed virtually the same frequency of hyper- and hypomethylated promoters in the two stages compared to WT, the status of hypermethylated promoters in *phyB1B2* increased over 60% from the IG to BK stage, while the number of loci with hypomethylation decreased 50% (Figure 2B; Supplementary Table 14). In contrast, *phyA* showed more hypermethylated promoters in the CHG context in the IG stage than in the BK stage, while their levels in *phyB1B2* mutant remained similar upon ripening (Figure 2B; Supplementary Table 15). In the CHH context, the number of hypermethylated promoters decreased in both genotypes from the IG to BK stages (Figure 2B; Supplementary Table 16).

These results indicate that PHY deficiency results in massive promoter hypermethylation in both the IG and BK stages of tomato fruit development. Moreover, they reinforce the role of PHYB1B2 in ripening-associated demethylation and its putative effect on gene expression.

### Effect of PHY-Mediated Differential Methylation on the Transcriptome

To assess whether the differential methylation of gene promoters affects mRNA levels, we compared data from DEGs and DMPs between genotypes. Supplementary Figure 4 shows scatter plots of promoter methylation vs. mRNA fold changes for comparisons of the two genotypes at the two examined developmental stages in the three mC contexts. The most evident result is that among the thousands of loci with identified DMPs (Figure 2B), only hundreds of the loci were also differentially expressed (Supplementary Table 17; 0.7% for IG *phyA*, 1.6% for IG *phyB1B2*, 5.6% for BK *phyA* and 7.4% for BK *phyB1B2*), raising an intriguing question about the biological significance of the extensive change in the methylation pattern observed in the mutants. In contrast, the percentages of the DEGs showing DMPs were 73% for IG *phyA*, 76% for IG *phyB1B2*, 72% for BK *phyA* and 75% for BK *phyB1B2*. Many more DEGs with DMPs were observed in BK than in IG fruits and in *phyB1B2* than in the *phyA* genotype (Figure 1C; Supplementary Figure 4). The functional categorization of these genes revealed a similar category distribution to the DEGs (Figure 1C; Supplementary Tables 18–21). At the IG stage, there were seven categories in which at least 2% of the loci showed DMPs and differential expression in both genotypes: photosynthesis, phytohormone action, RNA biosynthesis, protein modification and homeostasis, cell wall organization and solute transport, whereas *phyB1B2* additionally impacted lipid metabolism (Figure 1C). In the BK stage, the categories in which at least 2% of the DEGs showed DMPs were lipid metabolism, phytohormone action, RNA biosynthesis, protein modification and homeostasis, cell wall organization and solute transport-related functions in both genotypes, while *phyA* exclusively impacted carbohydrate metabolism and external stimuli, and *phyB1B2* exclusively affected photosynthesis, chromatin organization and cell cycle categories.

Interestingly, when comparing IG and BK stages, 42.5%, 34.2%, and 18.8% of the DMPs were associated with DEGs, while 79.5%, 76.6%, and 71.5% of the DEGs showed differences in promoter methylation in WT, *phyA* and *phyB1B2*, respectively, (Supplementary Figure 5). These results demonstrate that the altered mRNA profile of *phyA* and *phyB1B2* fruits are associated with marked changes in promoter methylation; however, massive genome-wide PHY-induced methylation reprogramming has a still uncharacterized role beyond the regulation of mRNA accumulation. Moreover, promoter methylation has a more significant effect on gene expression regulation during BK than in the IG stage (Supplementary Figure 4). Additionally, the data showed that PHYB1B2 has a more extensive influence on gene expression regulated *via* promoter methylation than PHYA, reinforcing the above conclusion that PHYB1B2 affects CG ripening-associated demethylation (Figures 2A,B; Supplementary Figure 4).

### The sRNAome Is Altered by PHY Deficiency

**Figure 3:**
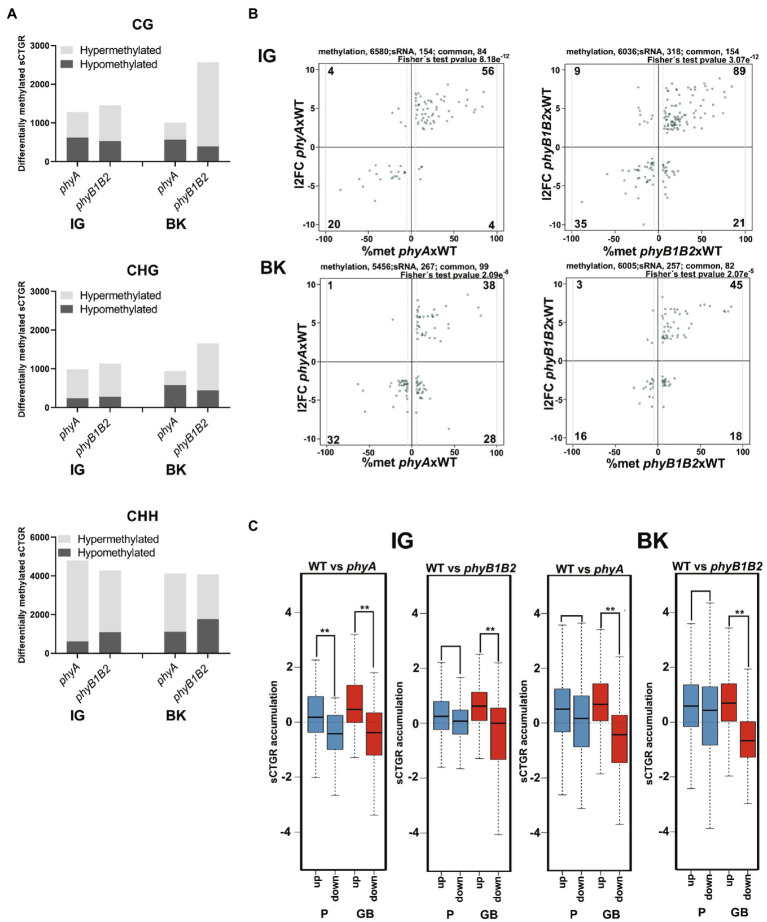
Phytochrome deficiency impacts the sRNAome profile. **(A)** Total number of differentially methylated sRNA cluster-targeted genome regions (sCTGRs). **(B)** Scatter plots show the relationship between the differential accumulation of cluster sRNAs and a minimum of 5% differential methylation of their sCTGRs. The result of Fischer’s test for the association of the two datasets is shown (*p* ≤ 2.07e^−5^). **(C)** Boxplots show changes in the accumulation of cluster sRNAs in promoter (P, 2 Kb upstream of the 5′ UTR end) and gene body (GB) regions for up- and downregulated DEGs. Asterisks indicate statistically significant differences by the Wilcoxon–Mann–Whitney test (^**^p < 0.0001). All results represent the comparison of *phyA* and *phyB1B2* to the wild type in immature green (IG) and breaker (BK) fruit stages.

To assess the involvement of RdDM in PHY-mediated transcriptome regulation, the sRNAome was analyzed in fruits at the IG and BK stages from both mutants and the WT genotype (Supplementary Table 4A). A total of 28,314 clusters of sRNAs were identified across the whole genome in at least one of the samples, including 7,984 in gene bodies, 7,863 in promoter regions, 7,966 in TEs and the remaining 4,501 across intergenic regions (Supplementary Figure 3; Supplementary Table 4B). The methylation level was evaluated for each sRNA cluster-targeted genomic region (sCTGR). Similarly, as observed for promoter regions, BK fruits from *phyB1B2* showed the highest number of hypermethylated sCTGRs within the CG and CHG symmetrical contexts. Moreover, the greatest number of differentially methylated sCTGRs was observed in the asymmetrical context CHH (Figure 3A; Supplementary Tables 4G–J).

sCTGR methylation levels and sRNA accumulation data were compared, and among a total of 154, 318, 267, and 257 differentially accumulated sRNA clusters (Supplementary Tables 4C–F), 84, 154, 99, and 82 also showed differential methylation in their targeted genomic region in *phyA* IG, *phyB1B2* IG, *phyA* BK and *phyB1B2* BK mutant fruits compared to WT, respectively, showing a strong association (*p* < 0.005) between the two datasets (Figure 3B; Supplementary Tables 4G–J). Intriguingly, this positive association was not observed in the transition from the IG to BK stages (Supplementary Figure 6), suggesting that the global methylation changes *via* RdDM could be attributed to PHY deficiency. Moreover, a clear alteration in sRNA accumulation was observed in *phyB1B2*, since almost no clusters with less sRNA accumulation were observed in BK compared to the IG stage (Supplementary Figure 6).

Further, we analyzed whether this association between sRNA accumulation and sCTGR methylation impacted gene expression levels. Notably, regardless of the fruit developmental stage, changes in the accumulation of sRNA located in gene bodies (GBs), and not in the promoter (P) region were positively correlated with the mRNA level (Figure 3C; Supplementary Tables 4K–N). Among these loci, two interesting examples were identified: the well-known ripening-associated genes *RIPENING INHIBITOR* (*RIN*, Solyc05g012020; Vrebalov et al., 2002) and *FRUITFULL2* (*FUL2*, Solyc03g114830; Bemer et al., 2012), which showed higher expression in *phyB1B2* at the IG stage (Supplementary Figure 7A) and higher sRNA accumulation and sCTGR methylation across their GBs (Supplementary Figure 7B) compared to WT. Altogether, these findings revealed: (i) impaired RdDM in BK fruits of *phyB1B2*, indicated by the absence of clusters with less sRNA accumulation (Supplementary Figure 6); and (ii) that GB RdDM is an important mechanism that positively regulates gene expression in a PHY-mediated manner during fruit development (Figure 3).

### PHYB1B2-Dependent Methylation Regulates Fruit Chlorophyll Accumulation

The categorization of DEGs associated with differential promoter methylation revealed a prominent representation of the photosynthesis category in the fruits of the *phyB1B2* mutant at the IG stage (Figure 1C). Among the 32 genes, 22 were downregulated and hypermethylated in the promoter region (Supplementary Tables 18 and 19). Most of these genes encode chlorophyll-binding proteins, structural photosystem proteins and chlorophyll biosynthetic enzymes. This might at least partly explain the reduction of 50% in the total chlorophyll level observed in *phyB1B2* IG fruits (Supplementary Figure 8A). The detailed analysis of the chlorophyll biosynthetic *PROTOCHLOROPHYLLIDE OXIDOREDUCTASE 3* (*POR3*, Solyc07g054210) and two *CHLOROPHYLL A/B BINDING PROTEINs* (*CBP*, Solyc02g070990 and *CAB-3c*, Solyc03g005780) genes showed that their reduced mRNA levels in *phyB1B2* (Supplementary Figure 8B) correlated with the presence of hypermethylated regions in the promoters (Supplementary Figure 8C). These results suggest that the transcription of genes involved in chlorophyll metabolism and the photosynthetic machinery in tomato fruits is affected by the PHYB1B2-dependent methylation status of their promoter regions.

### The Methylation-Mediated Regulation of Fruit Ripening Is Influenced by PHYB1B2 Signaling

**Figure 4:**
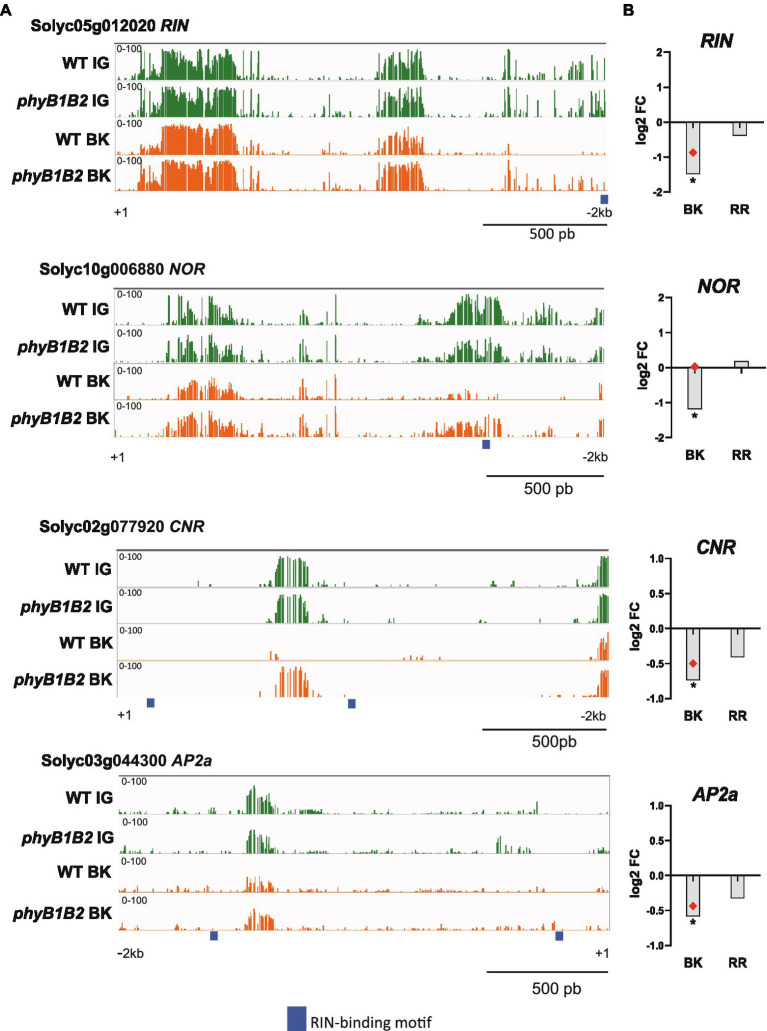
PHYB1/B2 influence on fruit ripening is associated to the promoter demethylation of master ripening-associated transcription factors. **(A)** Differentially methylated promoters of the *RIPENING INHIBITOR* (*RIN*), *NON-RIPENING* (*NOR*), *COLORLESS NON-RIPENING* (*CNR*) and *APETALA 2a* (*AP2a*) loci between the *phyB1B2* and WT genotypes. Green and orange indicate cytosine methylation levels in immature green (IG) and breaker (BK) fruits, respectively. Thick blue lines indicate RIN peak binding sites according to ChIP-seq data (Zhong et al., 2013). **(B)** Relative expression from the RT-qPCR assay of genes encoding master ripening transcription factors in BK and red ripe (RR) fruits from *phyB1B2*. Expression levels represent the mean of at least three biological replicates and are relative to WT. Asterisks indicate statistically significant differences by two-tailed Student’s *t*-test compared to WT (^*^p < 0.05). Red dots indicate data from RNA-seq in the same fruit developmental stage validated by RT-qPCR.

In their seminal study, Zhong et al. (2013) revealed that the extensive methylation in the promoter regions of ripening-associated genes gradually decreases during fruit development. Interestingly, RNA biosynthesis, which includes transcription factors, is the most abundant functional category among the DEGs that showed DMPs (Figure 1C). Thus, we examined a set of ripening-associated transcription factors: *RIN*, *NON-RIPENING* (*NOR*, Solyc10g006880; Mizrahi et al., 1976), *COLORLESS NON-RIPENING* (*CNR*, Solyc02g077920; Manning et al., 2006) and *APETALA2a* (*AP2a*, Solyc03g044300; Karlova et al., 2011). The evaluation of the promoter regions clearly showed that while their methylation level decreased from the IG to BK stage in the WT genotype, they remained highly methylated in *phyB1B2* (Figure 4A). The maintenance of high methylation levels in the promoters of these key regulatory genes at the onset of fruit ripening was highly correlated with their transcriptional downregulation at the BK stage (Figure 4B).

**Figure 5:**
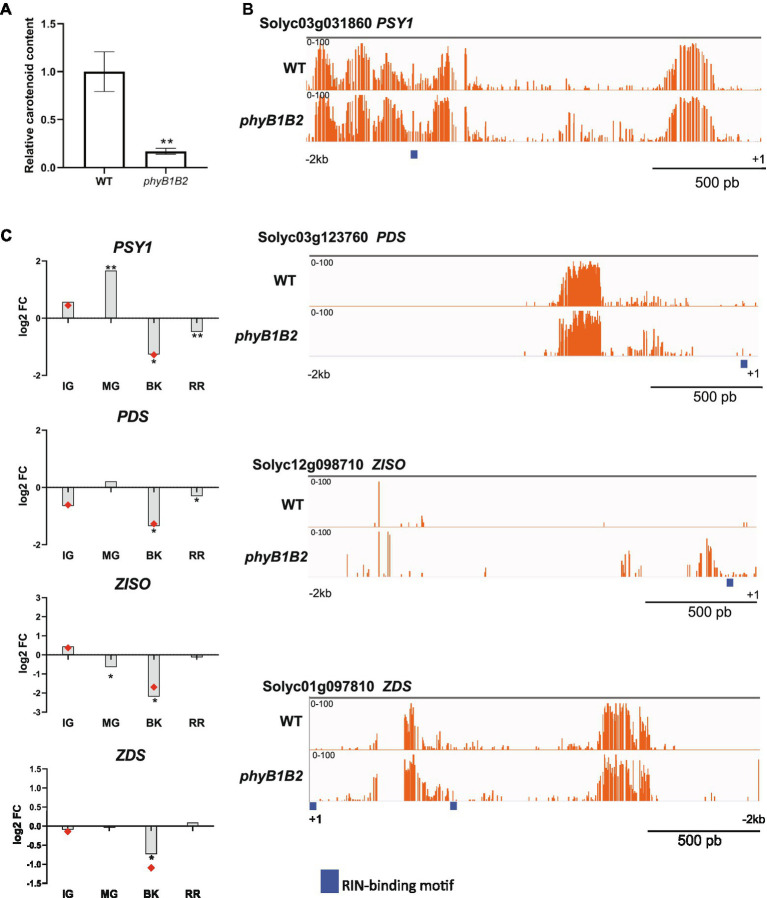
PHYB1/B2-dependent regulation of fruit carotenogenesis relies on the promoter demethylation of key carotenoid biosynthetic genes. **(A)** Relative contents of total carotenoids in red ripe (RR) fruits from *phyB1B2* and WT genotypes. Values represent the mean of at least three biological replicates. Asterisks indicate statistically significant differences by the two-tailed Student’s *t-*test between genotypes (^**^p < 0.01). **(B)** Differentially methylated promoter sites of the *PHYTOENE SYNTHASE 1* (*PSY1*), *PHYTOENE DESATURASE* (*PDS*), *15-CIS- ζ-CAROTENE* (*ZISO*) and *ZETA-CAROTENE DESATURASE* (*ZDS*) loci between the *phyB1B2* and WT genotypes. Orange bars indicate cytosine methylation levels in breaker (BK) fruits. Thick blue lines indicate RIN binding sites according to ChIP-seq data (Zhong et al., 2013). **(C)** Relative expression of carotenoid biosynthetic enzyme-encoding genes in immature green (IG), mature green (MG), BK and RR fruits from *phyB1B2* determined by RT-qPCR. Red dots indicate data from RNA-seq in the same stage. The expression levels represent the mean of at least three biological replicates and are relative to WT. Asterisks indicate statistically significant differences by the two-tailed Student’s *t-*test compared to WT (^*^p < 0.05 and ^**^p < 0.01).

Carotenoid accumulation is probably the most appealing and best-investigated trait of tomato fruits. In agreement with previous findings (Bianchetti et al., 2020), ripe *phyB1B2* fruits showed a 5-fold reduction in carotenoid content compared to WT (Figure 5A) which is in line with the observed reduction in the fruit color intensity (Chroma; Supplementary Figure 2D). To evaluate whether this effect is a consequence of the methylation-mediated regulation of carotenoid biosynthesis genes, we further analyzed the promoters of *PHYTOENE SYNTHASE 1* (*PSY1*, Solyc03g031860), *PHYTOENE DESATURASE* (*PDS*, Solyc03g123760), *15-CIS- ζ-CAROTENE* (*ZISO*, Solyc12g098710) and *ZETA-CAROTENE DESATURASE* (*ZDS*, Solyc01g097810), which, except for *PDS*, were hypermethylated in *phyB1B2* BK fruits (Supplementary Table 13). The mC profile confirmed the presence of hypermethylated regions in all four promoters (Figure 5B), which might explain the reduced mRNA levels of these genes observed in *phyB1B2* (Figure 5C).

RIN is one of the main TFs controling ripening-associated genes by directly binding to their promoters. RIN binding occurs in concert with the demethylation of its targets (Zhong et al., 2013). To examine whether RIN binding site methylation could be affected by the *phyB1B2* mutation in the ripening-related master transcription factors and carotenoid biosynthetic gene promoters, we mapped the available RIN ChIP-seq data (Zhong et al., 2013) and performed *de novo* motif discovery (Supplementary Figure 9). Interestingly, the levels of mCs around the RIN target genes promoters, *NOR*, *CNR*, and *AP2a*, were higher in the *phyB1B2* than in WT. Moreover, the *RIN* promoter itself was hypermethylated nearby the RIN binding site in *phyB1B2* BK fruits, suggesting a positive feedback regulatory mechanism (Figure 4A). Finally, in the *phyB1B2* mutant, the *PSY1*, *PDS*, *ZISO*, and *ZDS* promoters showed higher methylation overlapping with RIN target binding sites (Figure 5B), indicating that the upregulation of carotenoid biosynthesis genes during tomato ripening is dependent on the PHYB1B2-mediated demethylation of RIN target binding sites. Altogether, our findings showed that PHYB1B2 is a major player in fruit ripening by affecting the promoter demethylation of master transcriptional regulators and carotenoid biosynthesis genes.

### *Cis-*Regulatory PIFs/HYx/RIN Elements in Promoter Regions of *phyB1B2* DEGs

**Figure 6:**
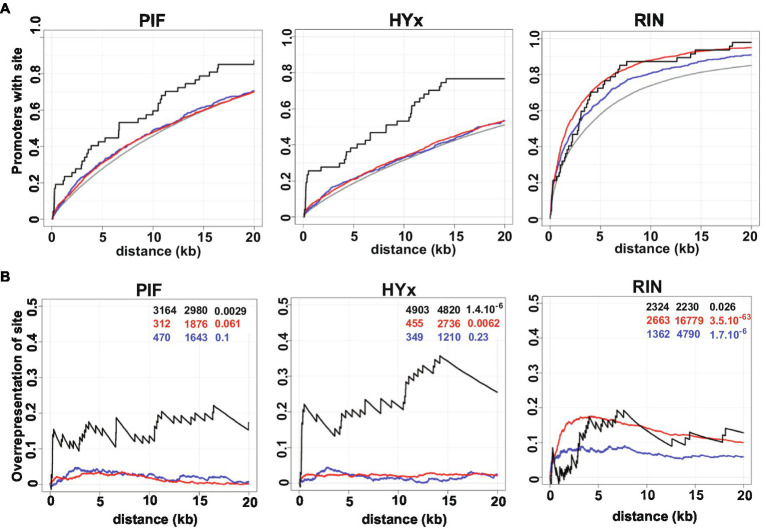
Positional distribution and enrichment of TF binding sites on PHYB1B2 regulated genes. The three gene dataset analyzed were: upregulated (red), downregulated (blue) and chromatin-remodeling (black) DEGs in *phyB1B2* at breaker stage. **(A)** Additive gene fraction harboring the indicated element in comparison with randomly chosen gene set (grey). **(B)** Over-representation of elements in the regulated genes in comparison to the randomly chosen gene set by subtracting the curves shown in **(A)**. The enrichment score, *z*-score and *p*-value for each class of TF are shown from left to right as inset. PIF includes PHYTOCHROME INTERACTING FACTOR 1,3,4,5 and 7 sites; HYx includes ELONGATED HYPOCOTYL 5 (HY5) and HY5 HOMOLOG (HYH) from Jaspar Database; RIN sites are based in the peak calling of ChIP-seq data (Zhong et al., 2013). X axis indicates upstream distance from the transcription start site (TSS).

The frequency and overrepresentation of PHY-downstream effectors, particularly PIFs and HYx (HY5 and HYH), and RIN binding motifs on *phyB1B2* DEGs promoter regions were evaluated. Three gene datasets were separately analyzed: *phyB1B2-*upregulated, *phyB1B2*-downregulated and those related to chromatin organization functional category. The proportion of promoters that contains each motif in the analyzed region is depicted in Figure 6A. The results revealed that the promoter region of the chromatin organization DEGs were overrepresented in PIFs and HYx binding motifs (Figure 6B). These results suggest that the effect of PHYB1B2 on the expression of the chromatin organization genes is mediated by the downstream effectors: PIFs and HYx. Moreover, RIN binding motif was overrepresented on the three gene datasets evaluated, being higher on the *phyB1B2-*upregulated genes (Figure 6B).

## Discussion

It has been proposed that ripening mechanisms, including genes and their epigenetic marks, have evolved from pre-existing pathways, which performed distinct functions in the ancestral angiosperms, that upon whole-genome duplications and further neofunctionalization have provided functional diversification (The Tomato Genome Consortium, 2012). In special, DNA methylation is a conserved epigenetic mark important for genome integrity, development, and environmental responses in plants (Lang et al., 2017). In tomato, the dynamic methylation pattern during fruit development has been demonstrated to be a critical ripening regulation mechanism (Zhong et al., 2013; Zuo et al., 2020). DNA demethylation, mainly in the CG context, triggers the activation of genes involved in ripening and is an absolute requirement for the regulation of gene expression leading to the pigment accumulation and ethylene synthesis (Zhong et al., 2013; Liu et al., 2015; Lang et al., 2017; Lü et al., 2018). Simultaneously, the dynamic epigenome during fruit development is strictly regulated by environmental cues (Zhong et al., 2013). The prevailing model establishes PHYs as major components involved coordinating fruit physiology with the ever-changing light and temperature environmental conditions (Alves et al., 2020; Bianchetti et al., 2020). Thus, we explored the link between fruit epigenome reprogramming and these well-established light and temperature sensors (Legris et al., 2016).

Our data clearly showed that *phyA* and *phyB1B2* deficiencies modified the epigenome profile through methylome and sRNAome reprogramming. In particular, PHY-mediated DMPs and GB methylation were associated with transcriptome alterations that affected tomato fruit development; thus, indicating that active PHYs regulate, at least in part, the ripening-associated demethylation previously reported (Zhong et al., 2013). However, the massive alteration of methylation patterns observed in *phy* mutants suggests the existence of a still unclear genome regulatory mechanism.

The *phyA* and *phyB1B2* mutants showed a positive correlation between cluster sRNA accumulation, target methylation in GB and mRNA levels. In angiosperms, GB methylation has been associated with constitutively expressed genes (Lu et al., 2015; Bewick and Schmitz, 2017); however, PHY deficiency, intriguingly, seems to deregulate this mechanism affecting the temporal expression of regulated genes. The *RIN* and *FUL2* examples analyzed here clearly showed that sRNA accumulation and methylation were mainly located near transposable elements (TEs; Supplementary Figure 7). It is known that the insertion of TEs within GB can disrupt gene expression; thus, methylation-mediated TE silencing and GB methylation are evolutionarily linked (Bewick and Schmitz, 2017). The enhancement of TE-associated DNA methylation in GB (Figure 3C) and the absence of clusters with less sRNA accumulation in BK compared to the IG stage in *phyB1B2* (Supplementary Figure 6) might be explained by the overexpression of canonical RdDM genes: Solyc12g008420 and Solyc06g050510 encode homologs of RNA-DEPENDENT RNA POLYMERASE (RDRP) and the associated factor SNF2 DOMAIN-CONTAINING PROTEIN CLASSY 1 (CLSY1), respectively, both of which were upregulated in BK fruits from *phyB1B2* plants (Supplementary Table 5). Similarly, Solyc09g082480 and Solyc03g083170, which were also upregulated in *phyB1B2* BK fruits, are homologs of *A. thaliana* RNA-DIRECTED DNA METHYLATION 1 (RDM1) and DEFECTIVE IN MERISTEM SILENCING 3 (DMS3), respectively. The protein products of these genes, together with DEFECTIVE IN RNA- DIRECTED DNA METHYLATION 1 (DRD1), form the DDR complex, which enables RNA Pol V transcription (Pikaard and Scheid, 2014). To our knowledge, this is the first report to associate PHY-mediated sRNA accumulation and DNA methylation with mRNA levels in plants.

Several pieces of evidence have shown that PHYB1B2 has a more substantial impact on tomato epigenome regulation than PHYA. For example, BK fruits from *phyB1B2* displayed (i) a large number of DEGs associated with chromatin organization (Figure 1C); (ii) overall promoter hypermethylation in the CG context (Figure 2B); (iii) the highest number of DEGs associated with DMPs (Supplementary Figure 4); and (iv) half the number of DMPs associated with DEGs between the IG and BK stages compared to the WT (Supplementary Figure 5). In order to understand how *phyB1B2* mutation resulted in this massive epigenomic alteration, we closely looked at the DEGs related to the chromatin organization functional category.

The chromomethylase *SlMET1L* (Solyc01g006100) (also referred to as *SlCMT3*; Gallusci et al., 2016) displays the highest transcript abundance in immature fruits, which declines towards the fully ripe stage (Cao et al., 2014). In line with the higher level of DNA methylation, our transcriptome analysis showed that *SlMET1L* was upregulated in *phyB1B2* BK fruits. Conversely, *SlROS1L* demethylase (Solyc09g009080; Cao et al., 2014) also referred as *SlDML1* (Liu et al., 2015), was also upregulated in *phyB1B2* BK fruits. Although it might seem contradictory at first glance, it has been reported that the *A. thaliana ROS1* gene promoter contains a DNA methylation monitoring sequence (MEMS) associated with a Helitron transposon, which is methylated by AtMET1, positively regulating *AtROS1* gene expression (Lei et al., 2015). Similarly, *SlROS1L* harbours two transposable elements within its promoter and showed a higher methylation level in *phyB1B2* than in the WT genotype, suggesting a similar regulatory mechanism in tomato (Supplementary Figure 10; Supplementary Table 17).

The tomato homolog of *A. thaliana DECREASED DNA METHYLATION 1* (*DDM1*, Solyc02g085390) showed higher mRNA expression in *phyB1B2* mutant BK fruits than in their WT counterparts. DDM1 is a chromatin remodeling protein required for maintaining DNA methylation in the symmetric cytosine sequence (Zemach et al., 2013), which can be associated with the CG context hypermethylation observed in *phyB1B2* (Figure 2A).

Several histone modifiers showed altered expression in BK fruits from the *phyB1B2* mutant (Supplementary Table 11). The methylation of lysine residues 9 and 27 on H3 is associated with repressed genes. Histone lysine methyltransferases are classified into five groups based on their domain architecture and/or differences in enzymatic activity (Pontvianne et al., 2010). The BK fruits of the *phyB1B2* mutant displayed three differentially expressed lysine methyltransferases: Solyc03g082860, an upregulated H3K27 Class IV homolog; and two H3K9 Class V homologs, Solyc06g008130 and Solyc06g083760, showing lower and higher expression than WT fruits, respectively. Histone arginine methylation is catalyzed by a family of enzymes known as protein arginine methyltransferases (PRMTs). Solyc12g099560, a *PRMT4a/b* homolog, was upregulated in *phyB1B2* BK fruits. Interestingly, in *A. thaliana*, PRMT4s modulate key regulatory genes associated with the light response (Hernando et al., 2015), reinforcing the link between the PHYB1B2 photoreceptors and epigenetic control. Finally, tomato histone demethylases have been recently identified. *SlJMJ6*, whose expression peaks immediately after the BK stage, has been characterized as a positive regulator of fruit ripening by removing the H3K27 methylation of ripening-related genes, and *SlJMJ6*-overexpressing lines show increased carotenoid levels (Li et al., 2020). *SlJMJC1* (Solyc01g006680), which exhibits the same expression pattern (Li et al., 2020), is downregulated in the *phyB1B2* mutant, suggesting that this gene might exhibit a similar regulatory function to its paralog, inducing ripening in a PHYB1B2-dependent manner (Figures 4, 5).

Histone deacetylation plays a crucial role in the regulation of eukaryotic gene activity and is associated with inactive chromatin (Zhang et al., 2018). Histone deacetylation is catalyzed by histone deacetylases (HDACs). Fifteen *HDAC*s were identified in the tomato genome (Zhao et al., 2015). Among these, *SlHDA10* (Solyc01g009120) and *SlHDT3* (Solyc11g066840) were found to be downregulated and upregulated in *phyB1B2* BK fruits, respectively. SlHDA10 is localized in the chloroplast, and its transcript is highly expressed in photosynthetic tissues (Zhao et al., 2015); whether SlHDA10 deacetylates chloroplast proteins by silencing photosynthesis-related genes remains to be determined. Although *SlHDT3* is mainly expressed in immature stages of fruit development and its expression declines with ripening, its silencing results in delayed ripening and reduced *RIN* expression and carotenogenesis (Guo et al., 2017). On the other hand, the expression level of *SlHDT3* is increased in ripening-deficient mutants such as *Nr* or *rin* (Guo et al., 2017). Our results showed that *phyB1B2* mutant fruits displayed higher expression of *SlHDT3* and reduced *RIN* transcript levels at the BK stage, suggesting reciprocal regulation between these two factors. Thus, in line with the evidence reported by Guo et al. (2017), we propose that during the IG stage, *SlHDT3* is highly expressed, contributing to the epigenetic inhibition of ripening. The reduction in *SlHDT3* expression towards BK releases DNA methylation that, in turn, upregulates *RIN* tunning ripening-related epigenetic reprogramming and contributes to explain the high methylation levels observed in the *phyB1B2* mutant (Figure 2).

## Conclusion

**Figure 7:**
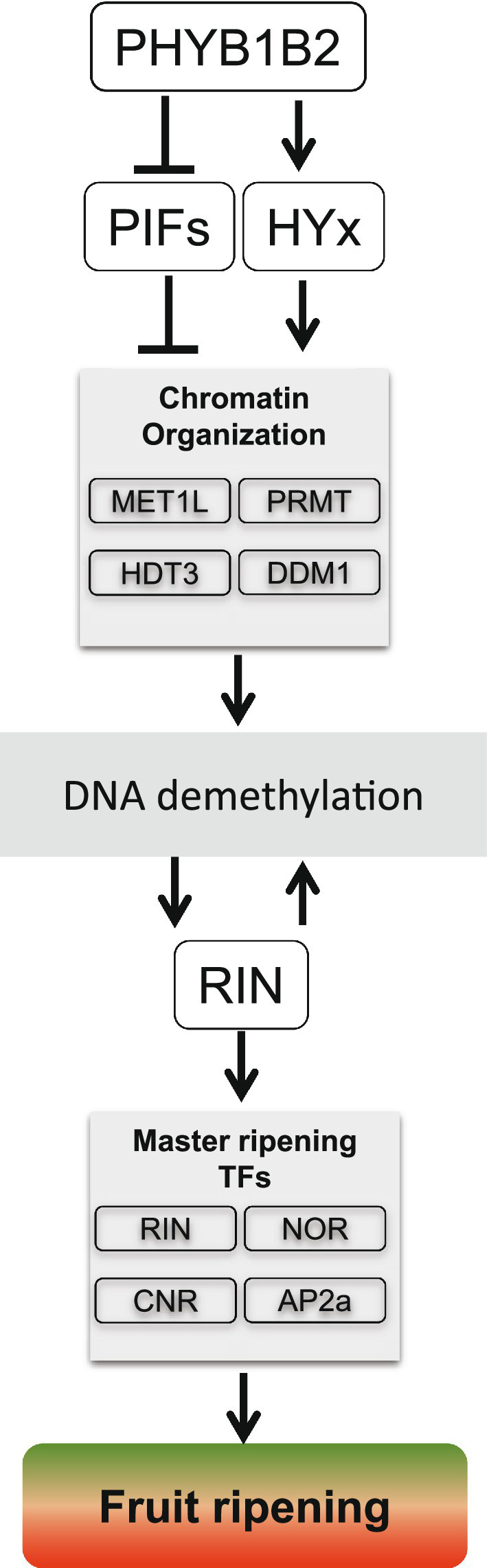
Conceptual model linking PHYB1B2 receptors, epigenetic mechanisms of gene expression regulation and fruit ripening. Active PHYB1B2, through the inactivation of PIFs and stabilization of HYx, regulates the expression of chromatin organization associated genes such as METL1, PRMT, HDT3, and DDM1, resulting in DNA demethylation and the induction of *RIN* ripening master TF expression. RIN targets include chromatin organization genes resulting in a positive feedback loop. Moreover, RIN enhances its own transcription, as well as other TFs (such as *NOR*, *CNR*, and *AP2a*) that finally induce a myriad of effectors triggering ripening.

Fruit ripening is a key trait for fitness and several alternative regulatory mechanisms guarantee the success of this process. This is most probably the reason why a single initiating signal has not been identified (Giovannoni et al., 2017). A complex interactive module involving DNA methylation level and tomato ripening- transcription factors was described (Zhong et al., 2013; Zuo et al., 2020). On the other hand, the link between chromatin remodeling and light signaling has been previously reported (Fisher and Franklin, 2011). Here, the comprehensive analysis of the experimental evidence allowed us to propose that PHYs, specially PHYB1B2, are important factors that participate in the crosstalk among chromatin organization and transcriptional regulators. The enrichment of PIF and HYx *cis*-regulatory motifs among the promoters of *phyB1B2-*DEGs associated with chromatin organization suggests that these PHY downstream factors regulate these genes that, in turn, trigger ripening-associated DNA demethylation. Epigenome reprogramming results in the adjustment of transcriptome including the induction of *RIN* master TF. The enrichment of hypermethylated RIN binding sites on the promoters of key ripening TFs (*CNR*, *NOR*, and *AP2a*), including *RIN* itself, in *phyB1B2*, indicates their RIN-mediated induction. These observations together with the fact that *rin* mutant is impaired in ripening-associated demethylation (Zhong et al., 2013), allow us to propose a positive regulatory loop between PHYs downstream effectors- and RIN-mediated DNA demethylation, driving the transcriptional regulation of ripening-associated TFs and, finally, to a shift in the expression profile along fruit development (Figure 7). The vast reservoir of data released here brings a new level of understanding about how epigenetic mechanisms orchestrate the response to PHY-mediated light and temperature fluctuations affecting important agronomical traits in fleshy fruits.

## Data Availability Statement

The datasets presented in this study can be found in online repositories. The names of the repository/repositories and accession number(s) can be found at: the datasets generated and/or analyzed during the current study are available in the Sequence Read Archive (SRA) under NCBI Bioproject PRJNA646733, with accession numbers SUB7763724, SUB7782168, and SUB7791358 for RNAseq, WGBS and small RNAseq, respectively (https://www.ncbi.nlm.nih.gov/bioproject/PRJNA646733).

## Author Contributions

RB performed most of the experiments and analyzed the data. LB, NB, LH, and RZ analyzed the data. DR performed the experiments. RB, LF, MR, and LB conceived the project, designed the experiments and wrote the paper, which was revised and approved by all authors. LB agrees to serve as the author responsible for contact and ensures communication. All authors contributed to the article and approved the submitted version.

## Funding

This work was supported by FAPESP (Fundação de Amparo à Pesquisa do Estado de São Paulo, grant number #2016/01128-9); RB was a recipient of a FAPESP fellowship (#2017/24354-7).

## Conflict of Interest

The authors declare that the research was conducted in the absence of any commercial or financial relationships that could be construed as a potential conflict of interest.

## Publisher’s Note

All claims expressed in this article are solely those of the authors and do not necessarily represent those of their affiliated organizations, or those of the publisher, the editors and the reviewers. Any product that may be evaluated in this article, or claim that may be made by its manufacturer, is not guaranteed or endorsed by the publisher.

## Footnotes

^1^ http://www.bioinformatics.babraham.ac.uk/projects/fastqc/

^2^ http://www.infostat.com.ar

^3^ https://www.ncbi.nlm.nih.gov/bioproject/PRJNA646733
