# Supplementary material for: Phytochrome-Mediated Light Perception Affects Fruit Development and Ripening Through Epigenetic Mechanisms
Source: Front Plant Sci. 2022 Apr 28;13:870974. doi: 10.3389/fpls.2022.870974 (PMC9096621; doi:10.3389/fpls.2022.870974)
Supplement: Supplementary file 19 [file Image_1.pdf]

SUPPLEMENTARY FIGURES

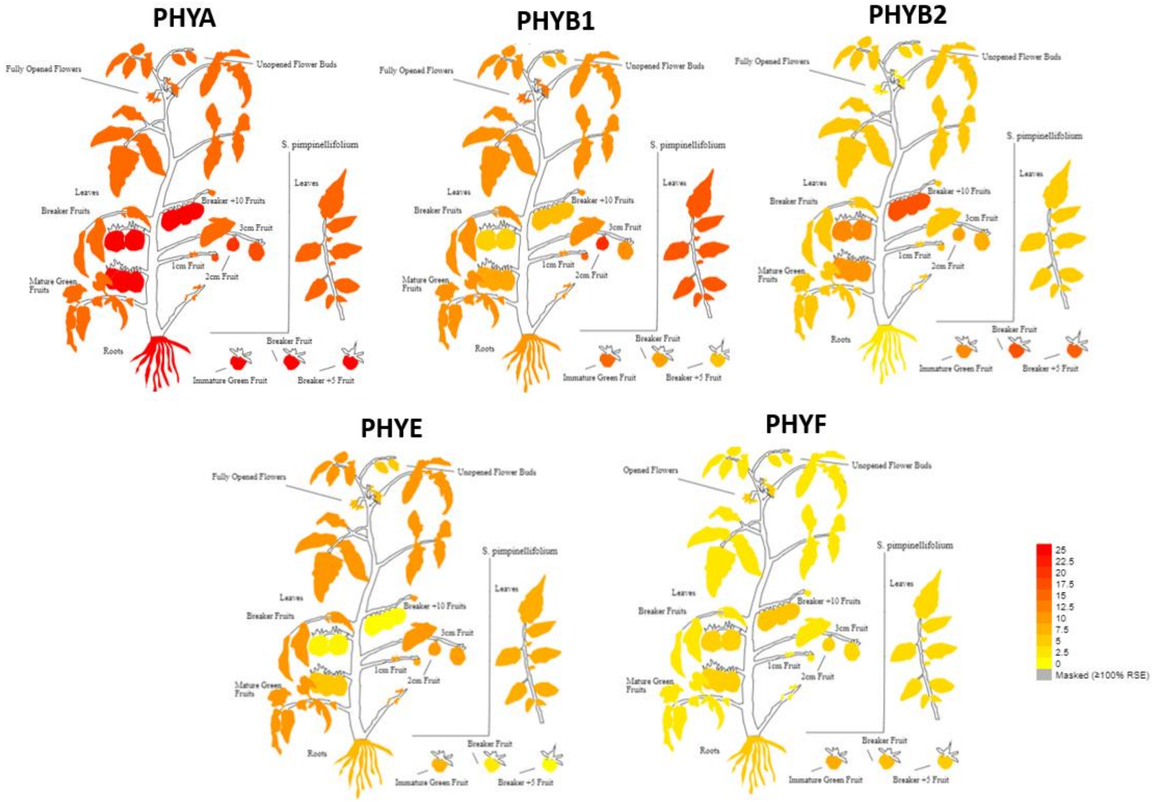

Supplementary Figure 1. Expression profile of the five-PHY encoding genes; *PHYA*, *PHYB1*, *PHYB2*, *PHYE* and *PHYF* in tomato (*Solanum lycopersicum* cv. Heinz). Available in the ePLANT PUBLIC DATABASE - http://bar.utoronto.ca/eplant_tomato/.

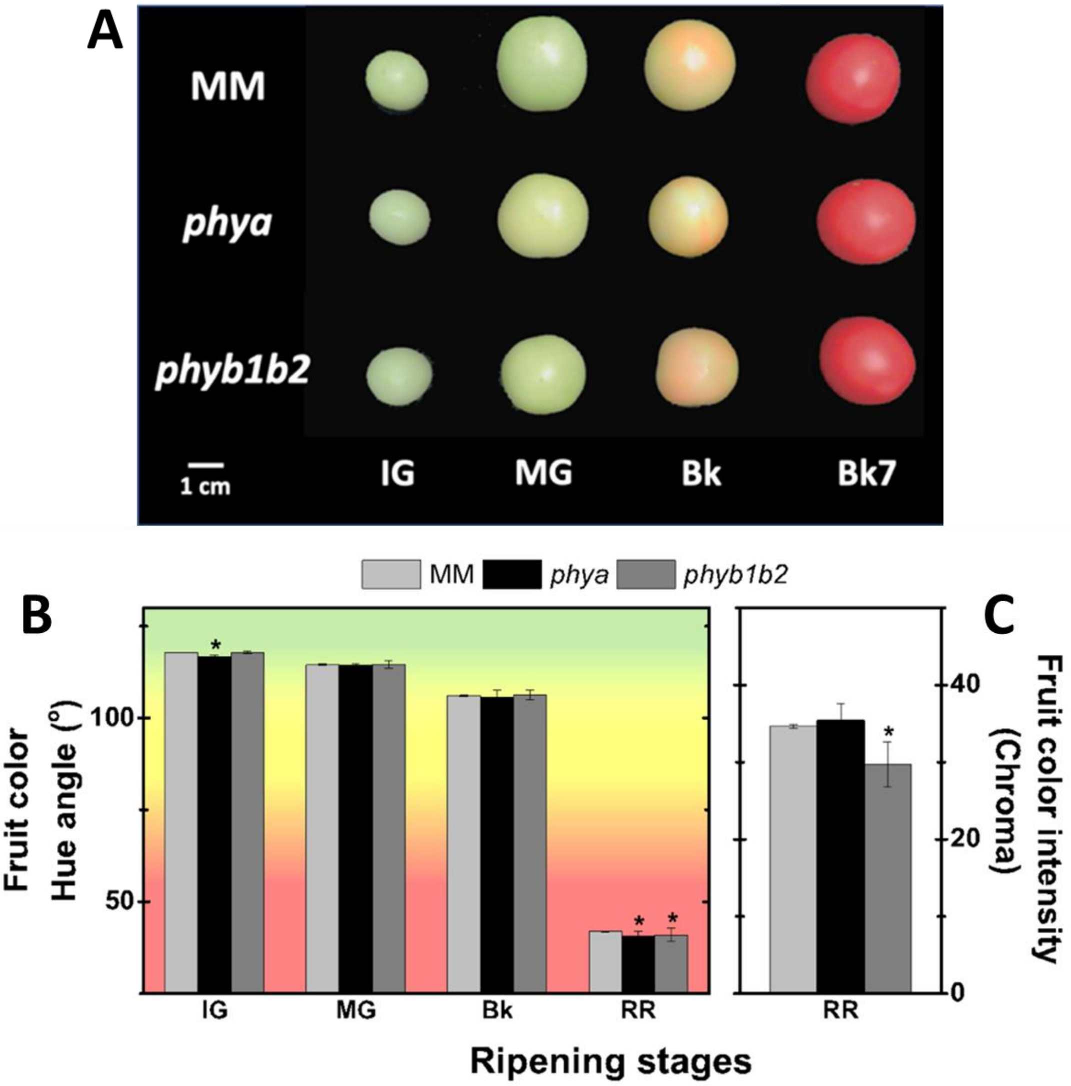

Supplementary Figure 2. Phenotype of collected fruits from tomato WT plants and *phyA* and *phyB1B2* knockout mutants (A) Developmental stages sampled. (B) Fruit color changes according to Hue angle. (C) Fruit color intensity (Chroma). *Statistically significant by two-tailed T-test < 0.05.

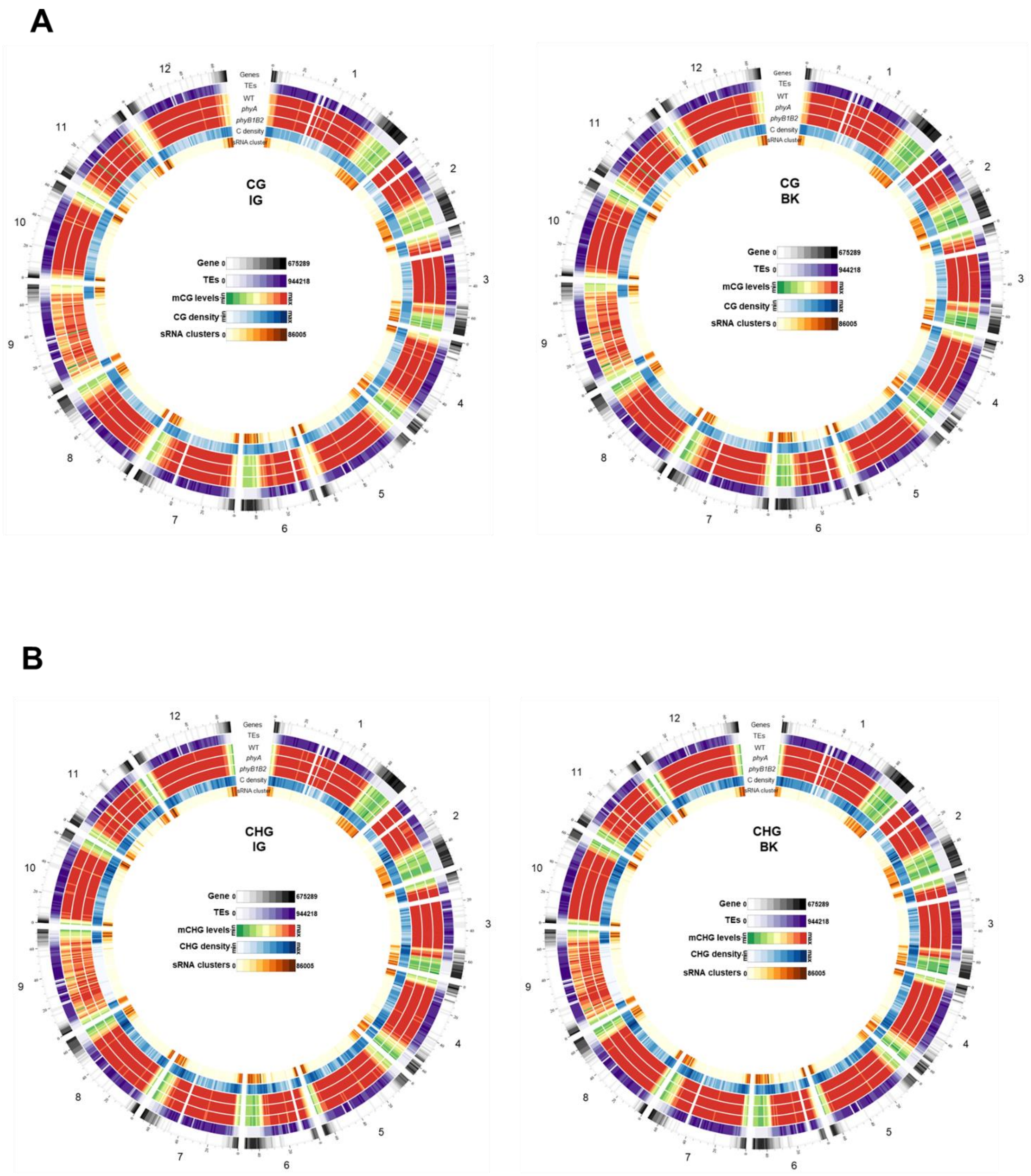

A
Genes
TEs
WT
phyA
phyB1B2
C density
sRNA cluster
CG
IG
Gene 0 675289
TEs 0 944218
mCG levels min max
CG density min max
sRNA clusters 0 86005
CG
BK
B
CHG
IG
Gene 0 675289
TEs 0 944218
mCHG levels min max
CHG density min max
sRNA clusters 0 86005
CHG
BK
1
2
3
4
5
6
7
8
9
10
11
12

C

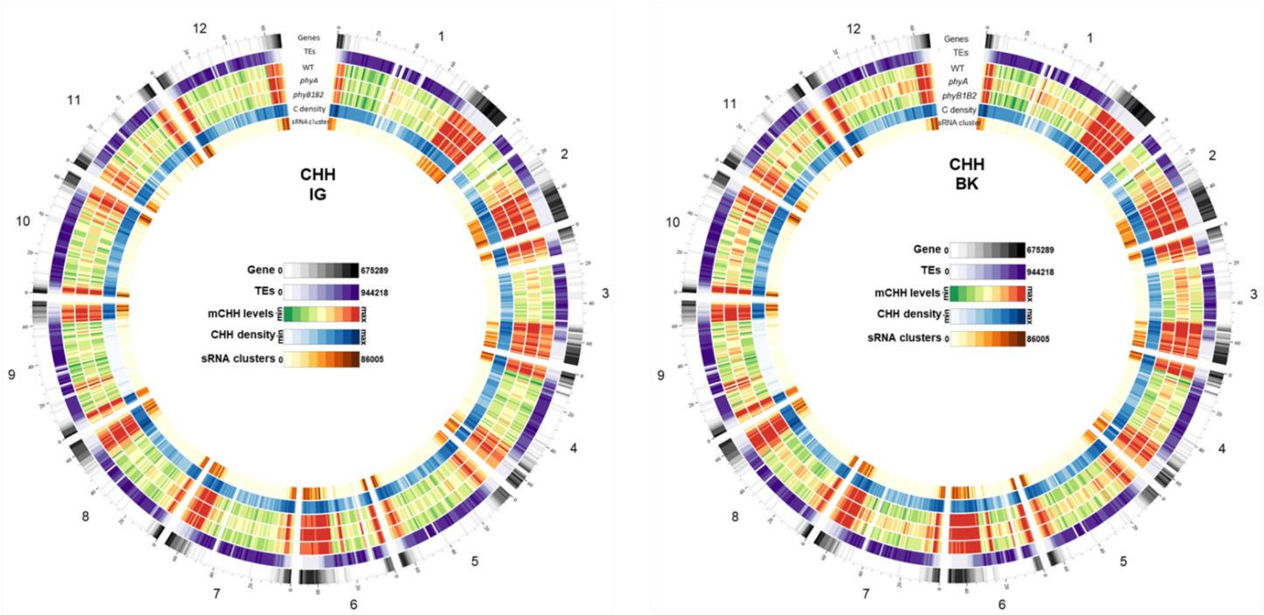

Supplementary Figure 3. Global methylation status in *phyA*, *phyB1B2* and WT at IG and BK stage for mCG (A), mCHG (B) and mCHH (C) contexts. Density plot of genes, transposable elements (TEs) and sRNAs clusters were estimated by the number of nucleotides covered per million. Methylation levels for CG, CHG and CHH contexts, are 40-90%; 25-80% and 10-30%, respectively. Cytosine density for CG, CHG and CHH contexts, are 0 - 17,488; 0 - 13,105 and 0 - 103,903 per million, respectively.

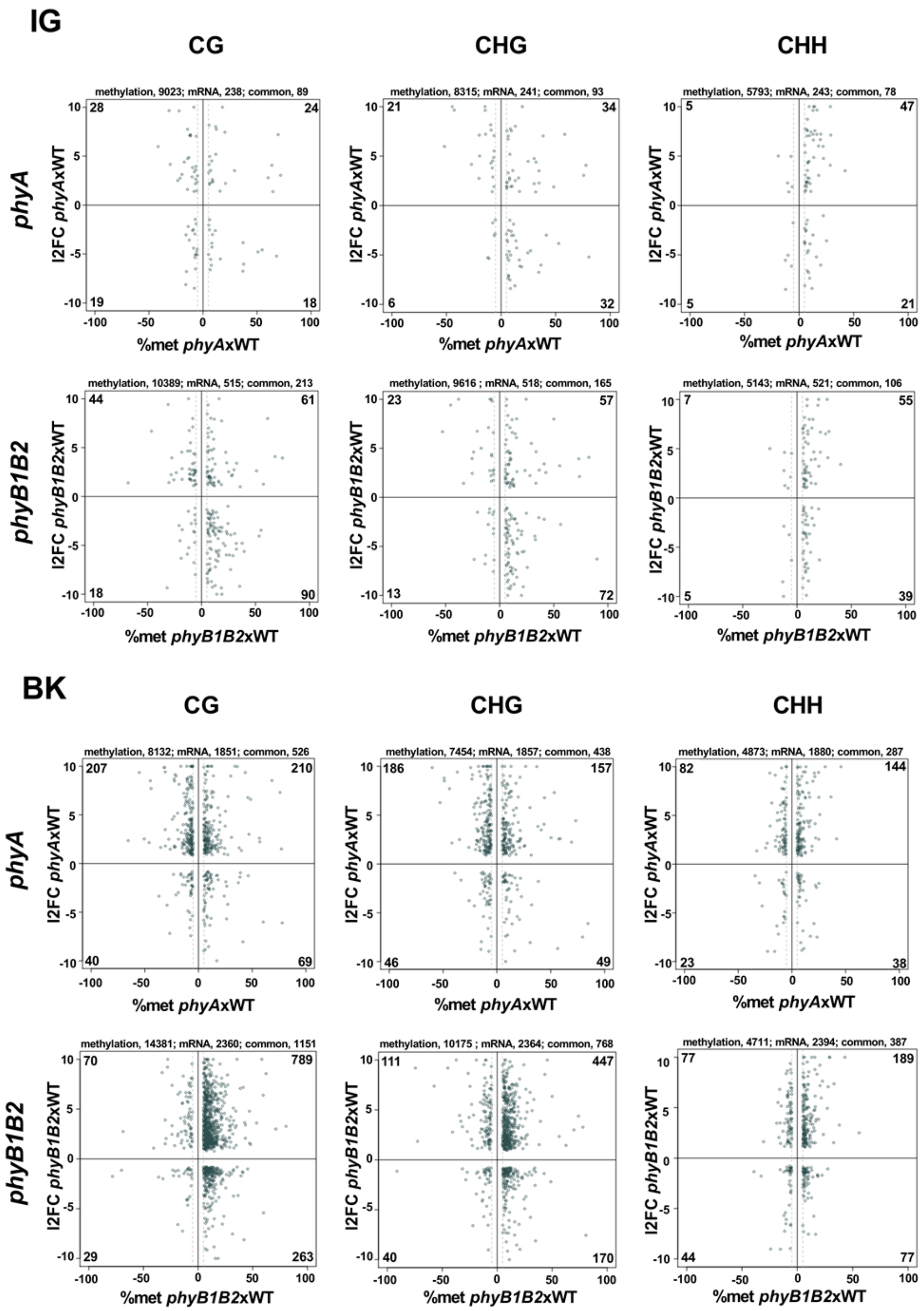

Supplementary Figure 4. mRNA level alterations associated with differences in the promoter methylation in *phyA* and *phyB1B2* compared to WT. Scatter plots show the DEGs that showed DMPs at immature green (IG) and breaker (BK) fruit stages, the axes indicate the variation between both parameters compared to WT(DEGs, FDR < 0.05; DMPs, FDR < 0.05) in CG, CHG and CHH contexts. Only DMPs with changes in methylation levels > 5% are shown.

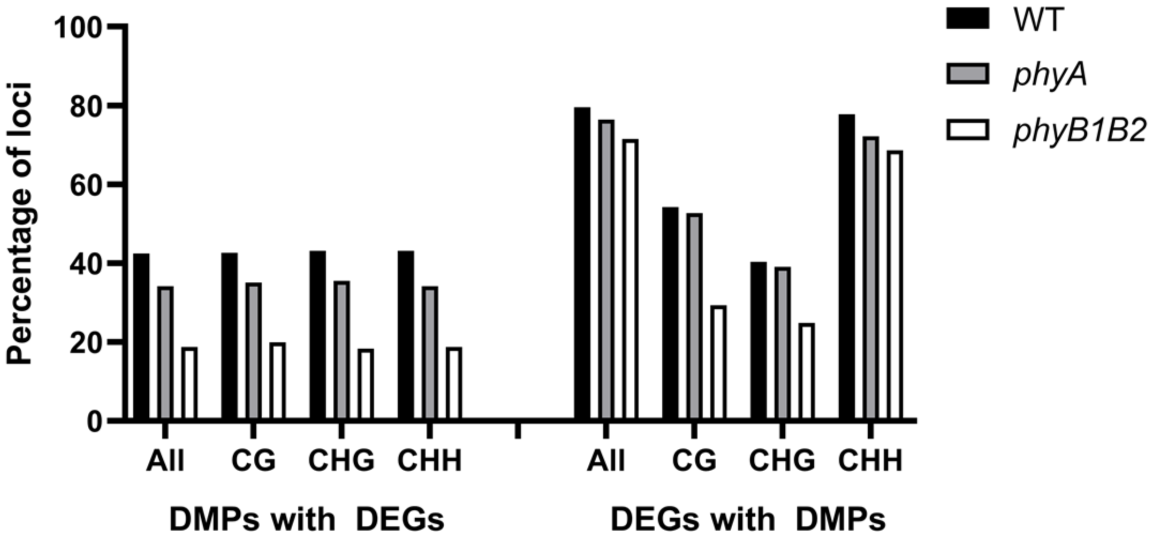

Supplementary Figure 5. Comparison of the association of DMPs and DEGs between immature green and breaker stages within each genotype. DMPs: differentially methylated promoters; DEGs: differentially expressed genes.

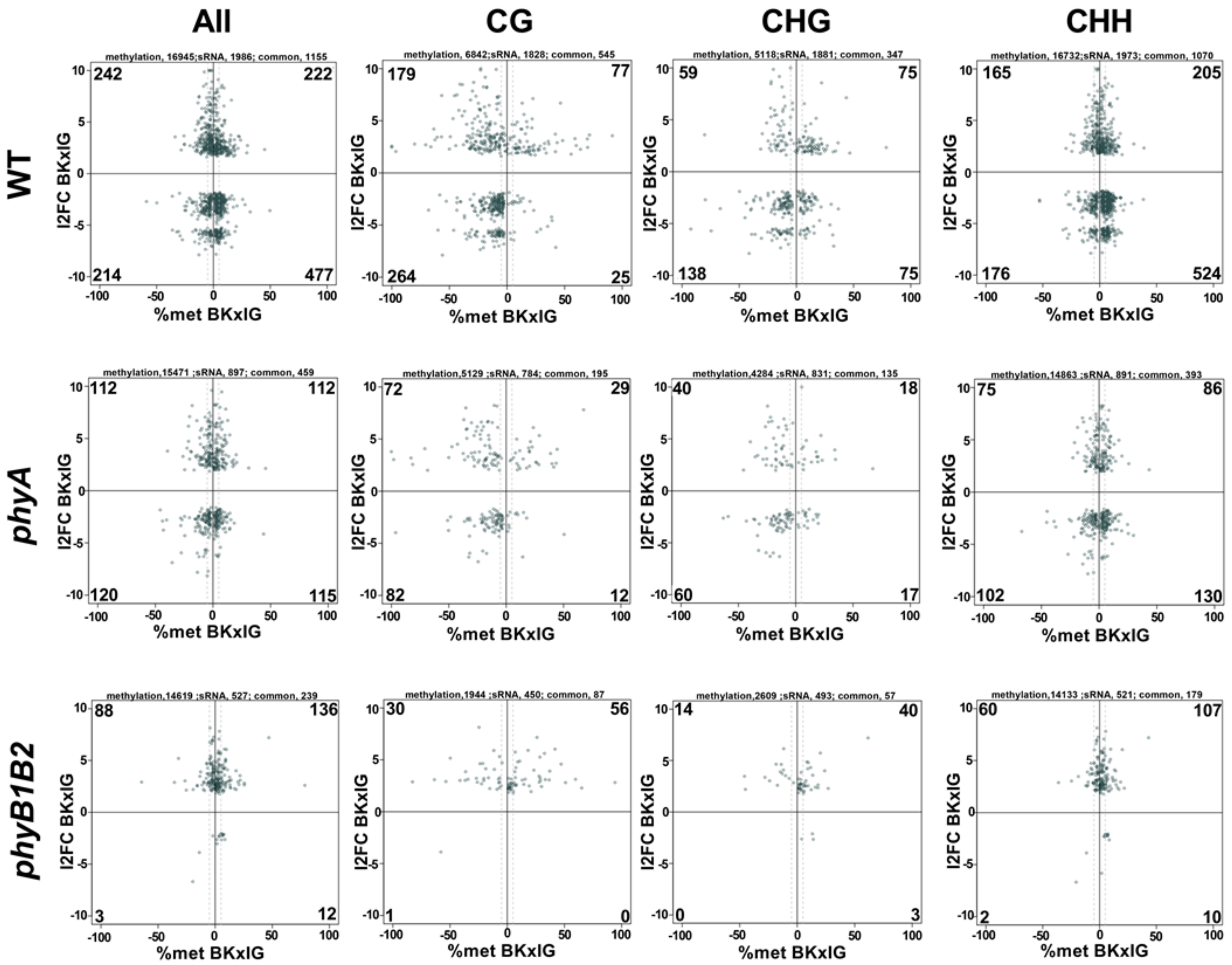

Supplementary Figure 6. Methylation levels in sRNA cluster targeted genomic region (sCTGR) and small RNA accumulation changes between IG and BK stages in WT, *phyA* and *phyB1B2* genotypes. Scatter plots show the relationship between the small RNA accumulation and differential methylation on their target genomic regions between immature green (IG) and breaker (BK) fruit stages.

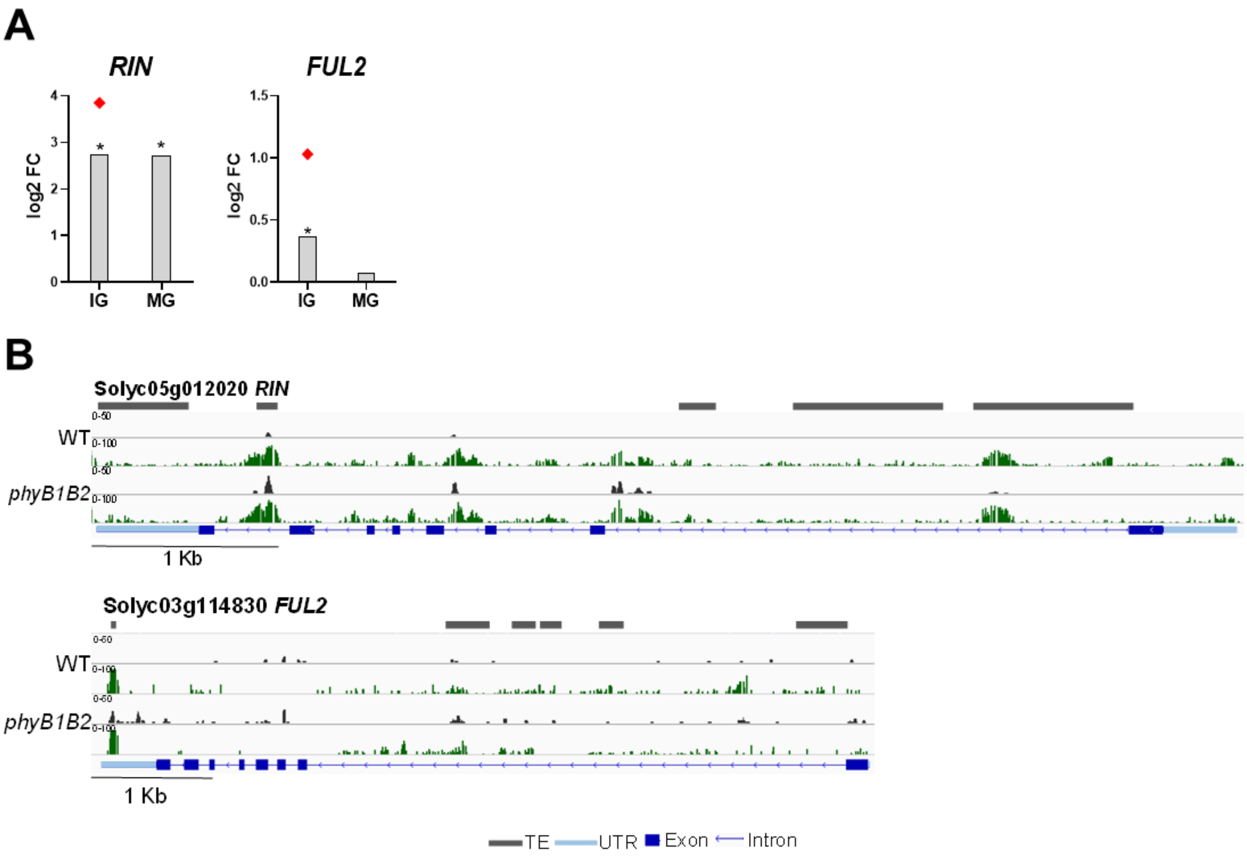

Supplementary Figure 7. Methylation across gene body regions differentially affects gene expression. (A) Relative expression of *RIPENING INHIBITOR* (*RIN*) and *FRUITFULL 2* (*FUL2*) in immature green (IG) and mature green (MG) fruits from *phyB1B2* determined by RT-qPCR. Red dots indicate data from RNA-seq in the same stage. Expression levels represent the mean of at least three biological replicates and are relative to the wild type (WT). Asterisks indicate statistically significant differences by the two-tailed Student´s *t* test compared to WT (* $p<0.05$). (B) Gene body methylation levels (green bars) and sRNA accumulation (black bars) within *RIN* and *FUL2* in IG fruits from the *phyB1B2* and WT genotypes.

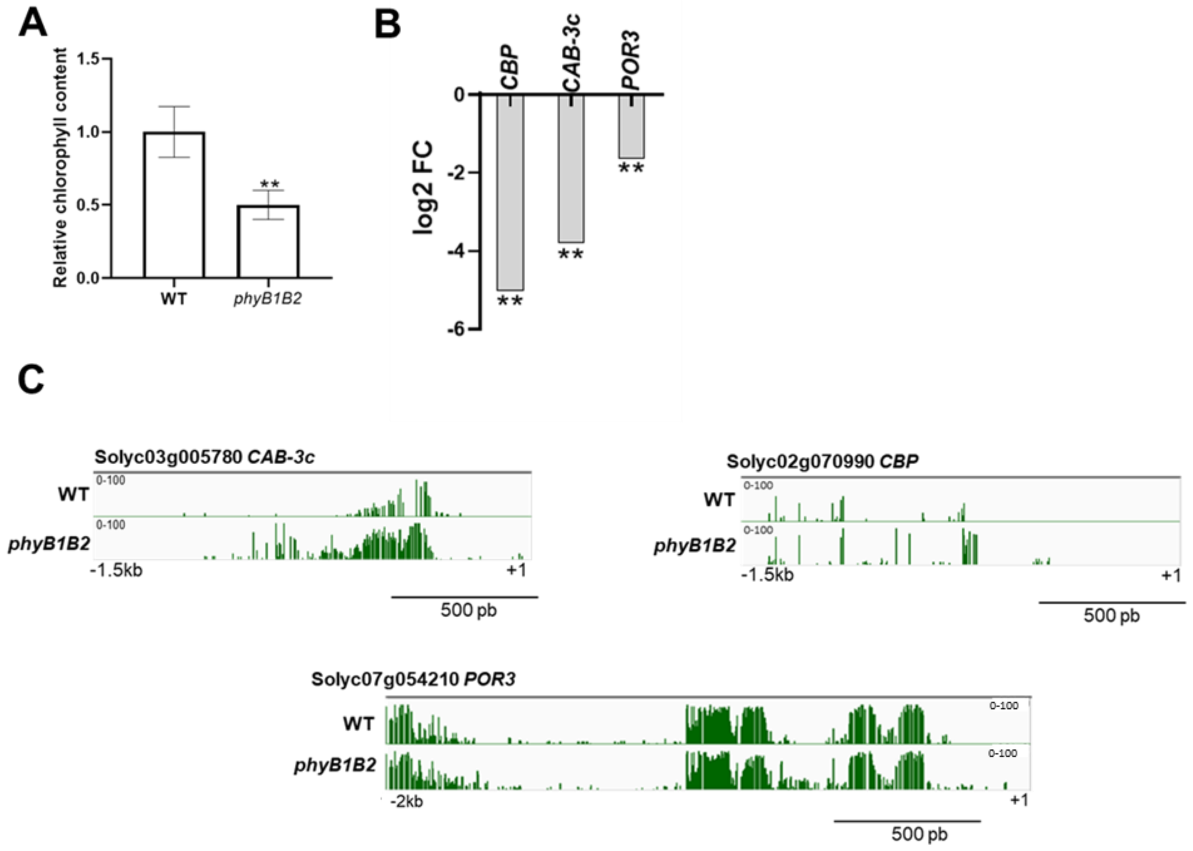

Supplementary Figure 8. PHYB1/B2-dependent methylation regulates fruit chlorophyll contents. (A) Relative content of total chlorophyll in IG fruits from *phyB1B2* and WT genotypes. Values represent the mean of at least three biological replicates. Asterisks indicate statistically significant differences by the two-tailed Student's *t* test between genotypes (** $p<0.01$). (B) Relative expression of *CHLOROPHYLL A/B BINDING PROTEINs* (*CBP* and *CAB-3c*) and *PROTOCHLOROPHYLLIDE OXIDOREDUCTASE 3* (*POR3*) in IG fruits from *phyB1B2* determined by RNA-seq. Expression levels represent the mean of at least three biological replicates and are relative to WT. Asterisks indicate statistically significant differences compared to WT (* FDR≤0.05). (C) Differential promoter (up to 2Kb upstream of TSS +1) methylation in *CBP*, *CAB-3c* and *POR3* in IG fruits from the *phyB1B2* and WT genotypes.

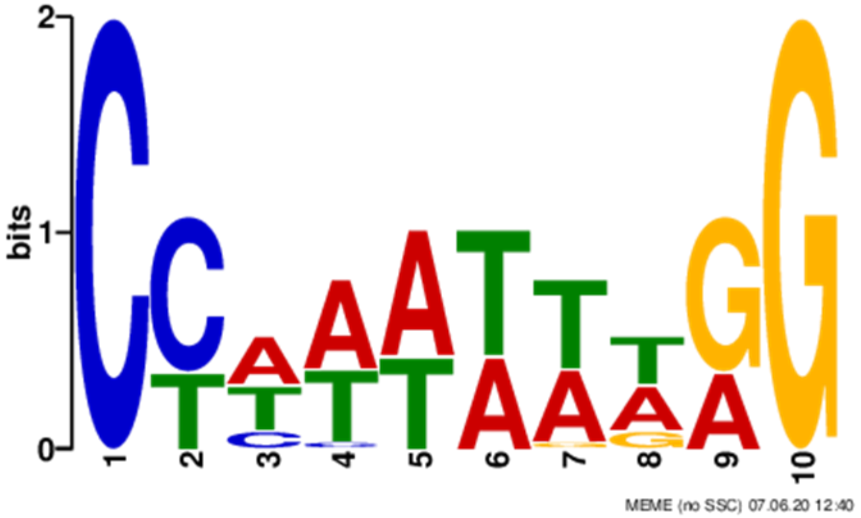

Supplementary Figure 9. RIN motif *de novo* discovered using MEME algorithm. Consensus sequence CCWWWWWWGG (CC(6W)GG) and extended TTWCCWWWWWWWGGWAA length=16.

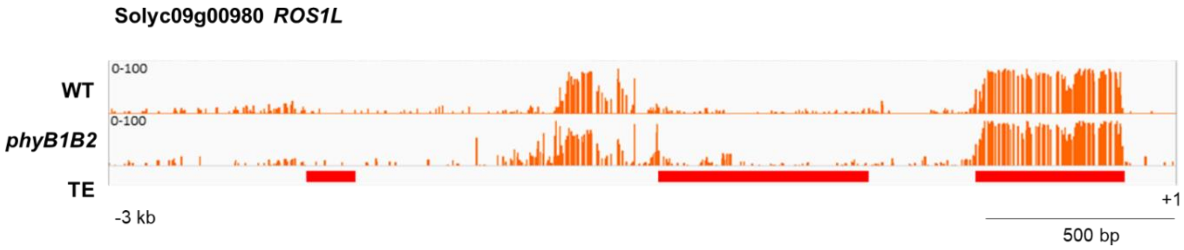

Supplementary Figure 10. DMP sites in the *ROS1L* locus in WT and *phyB1B2* BK fruits. Red rectangles indicate the presence of transposable element (TE).
